# Using machine learning to model older adult inpatient trajectories from electronic health records data

**DOI:** 10.1016/j.isci.2022.105876

**Published:** 2022-12-24

**Authors:** Maria Herrero-Zazo, Tomas Fitzgerald, Vince Taylor, Helen Street, Afzal N. Chaudhry, John R. Bradley, Ewan Birney, Victoria L. Keevil

**Affiliations:** 1 European Molecular Biology Laboratory, European Bioinformatics Institute (EMBL-EBI), Wellcome Genome Campus, Hinxton, Cambridgeshire CB10 1SD, UK; 2 Department of Medicine for the Elderly, Addenbrooke’s Hospital, Cambridge University Hospitals NHS Foundation Trust, Hills Road, Cambridge CB2 0QQ, UK; 3 Cambridge Clinical Informatics, Addenbrooke’s Hospital, Cambridge University Hospitals NHS Foundation Trust, Hills Road, Cambridge CB2 0QQ, UK; 4 Research and Development, Cambridge University Hospitals NHS Foundation Trust, Hills Road, Cambridge CB2 0QQ, UK; 5 Department of Medicine, University of Cambridge, Addenbrooke’s Hospital, Hills Road, Cambridge CB2 0QQ, UK; 6 NIHR Cambridge Biomedical Research Centre, Cambridge Biomedical Campus, Cambridge CB2 0QQ, UK

**Keywords:** Health technology, Diagnostic technique in health technology, Applied computing in medical science, Machine learning

## Abstract

Electronic Health Records (EHR) data can provide novel insights into inpatient trajectories. Blood tests and vital signs from de-identified patients’ hospital admission episodes (AE) were represented as multivariate time-series (MVTS) to train unsupervised Hidden Markov Models (HMM) and represent each AE day as one of 17 states. All HMM states were clinically interpreted based on their patterns of MVTS variables and relationships with clinical information. Visualization differentiated patients progressing toward stable ‘*discharge-like*’ states versus those remaining at risk of inpatient mortality (IM). Chi-square tests confirmed these relationships (two states associated with IM; 12 states with ≥1 diagnosis). Logistic Regression and Random Forest (RF) models trained with MVTS data rather than states had higher prediction performances of IM, but results were comparable (best RF model AUC-ROC: MVTS data = 0.85; HMM states = 0.79). ML models extracted clinically interpretable signals from hospital data. The potential of ML to develop decision-support tools for EHR systems warrants investigation.

## Highlights

• Time-series blood test & vital sign data from older inpatients were presented to HMM
• Hidden clinically interpretable states were extracted, linked with diagnoses and death
• States modeled inpatient trajectories, differentiating risk from admission-discharge
• The clinical interpretation of HMM states helped explain how ML models organize data

## Graphical abstract

**Figure:**
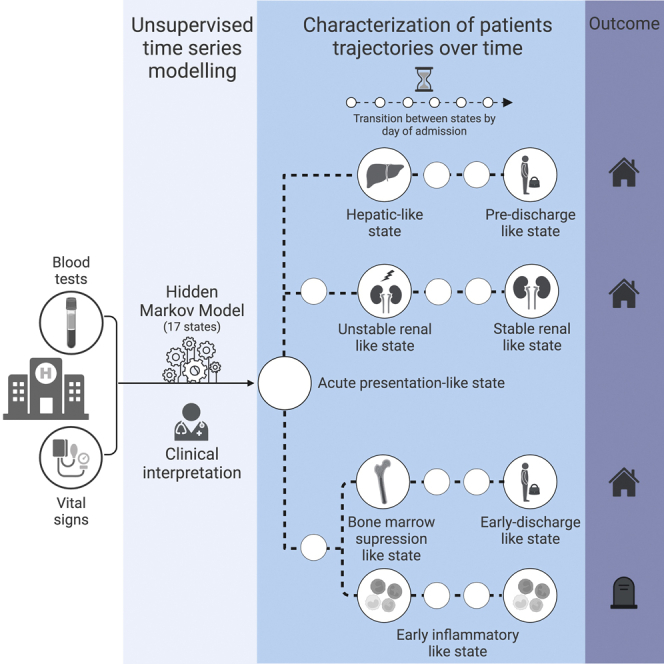

## Introduction

The growing implementation of Electronic Health Records (EHR) in National Health Service (NHS) hospitals can enhance the efficiency and safety of healthcare^1^ and provide large, detailed longitudinal datasets for clinical research.^2^ Machine Learning (ML) methods have been used to unravel hidden relationships within this complex ‘big data’ and, in selected settings, hospital EHR data have been used to augment development of stratified medicine, automated medical image analysis, and the prediction of clinical diagnoses and outcomes.^3^ However, its potential to transform healthcare has not yet been realised.^4^

Barriers to progress include the limited availability of research ready hospital EHR datasets. Data is collected primarily to support clinical care and challenges arise over patient confidentiality, data security, data quality, and the heterogeneity of data type, scale, frequency, regularity, and missingness.^3^^,^^4^^,^^5^ For example, whereas hospital EHR data is irregular with higher proportions of missingness than traditional research datasets, most ML techniques require complete datasets^6^ and benefit from a regularized representation of the raw data before model training.^7^^,^^8^^,^^9^ Furthermore, clinicians need to understand model outputs and the perceived ‘black box’ of some ML methods has been one barrier to clinical translation.^7^ Thus, successful representations of EHR data need to address the interpretability of ML models as well as their performance on prediction tasks.^10^^,^^11^^,^^12^

Considering this landscape, we formed a collaboration between clinicians, data scientists, research governance experts and clinical informatics specialists. We extracted de-identified data pertaining to >10,000 older adults admitted as an emergency to an NHS hospital, with the following aims. First, we establish if routinely collected healthcare data from this heterogeneous inpatient population can be processed into a research ready dataset suitable for longitudinal ML analyses. Specifically, we focus on 23 commonly but irregularly measured blood tests and vital signs data, used to monitor illness acuity and disease severity during inpatient admission episodes (AE). Second, we explore the application of unsupervised Hidden Markov Models (HMM) to this multivariate time-series (MVTS) data. HMM is a generative ML technique successfully used in other work to represent disease trajectories, in terms of progression through different disease states.^11^^,^^13^^,^^14^^,^^15^ An advantage of the HMM approach is that the state spaces generated can be retrospectively interrogated, allowing visualization of how the HMM organizes data and detailed clinical interpretation of model outputs. Thus, we aim to discover if HMM can uncover hidden, clinically interpretable signals from longitudinal blood test and vital signs data and provide novel insights into older adult inpatient trajectories, as well as facilitating clinician understanding of ML models. Finally, we evaluate the prediction of hospital outcomes using both the MVTS data and HMM states as input data to train Logistic Regression and Random Forest models, considering potential future applications of ML in the development of clinical decision support tools, fit for a digital healthcare age.

## Results

**Figure 1:**
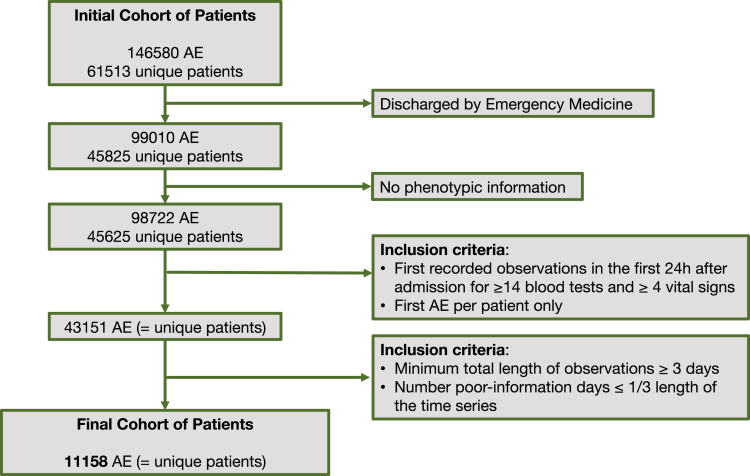
Description of inclusion criteria to select the final cohort of patients Phenotypic information includes age, sex, Clinical Frailty Scale score, and admission diagnosis. ‘*Rich information’* days were defined as days with information for ≥14 blood tests and ≥4 vital signs. All other days were defined as ‘*Poor Information’* days. AE, Admission episode.

The final dataset comprised 11,158 unique AEs (Figure 1). This dataset was divided into ‘training and validation’ (n = 8926) and ‘hold-out test’ datasets (n = 2232) and patient characteristics in both are described in Table S1. In the ‘training and validation’ dataset 47.2% of patients were women and 40.7% were aged 65–74 years with 59.3% aged >75 years.

### HMM states: Findings and clinical interpretation

**Figure 2:**
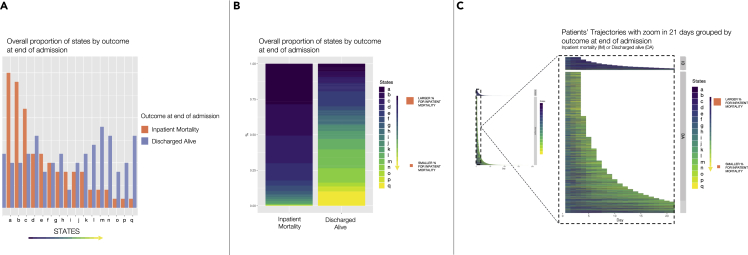
Visualization of the association between states and outcome at the end of admission (A) Relative frequency distribution of the 17 HMM states (labeled ‘a’ to ‘q’) after each patient day of each admission episode is assigned one state. The distribution is grouped by hospital outcome at the end of admission (either inpatient mortality [IM] or discharged alive [DA]). (B) Distribution of HMM states grouped by hospital outcome (IM or DA) with states represented by a color gradient based on the relative frequency of the state on the inpatient mortality group, as shown in (A) (darker colors for higher frequency on the inpatient mortality group and lighter colors for lower frequency). (C) Representation of patients’ trajectories from admission to discharge. Each day of each admission episode is represented by an HMM state, with states ‘a’ through to ‘q’ colored as in (B). The figure zooms in on the first 21 days of admission.

An unsupervised HMM was trained using expectation maximization with only the MVTS as input data so that each patient day of each AE was represented by one of 17 HMM states, instead of 23 numeric values for blood test and vital signs data. The distribution and overall proportion of these states are shown in Figure 2. Figure 2A demonstrates that the distribution of states is different for AEs ending in discharge alive compared to those ending with IM. Similarly, Figure 2B shows that some states are more common in patients discharged alive (‘yellow states’ e.g., state q) and others in patients who die during the AE (‘blue states’ e.g., state a). Figure 2C then visualizes the first 21 days of all AEs, with trajectories represented by the different states. AEs are again grouped by IM and trajectories visually differ depending on the final hospital outcome. As AEs progress, patients who experience IM transition into states more strongly associated with death (darker ‘blue’ states), whereas those not experiencing IM have states associated with being discharged alive enriched toward the end of their AEs (lighter ‘green/yellow’ states).

**Figure 3:**
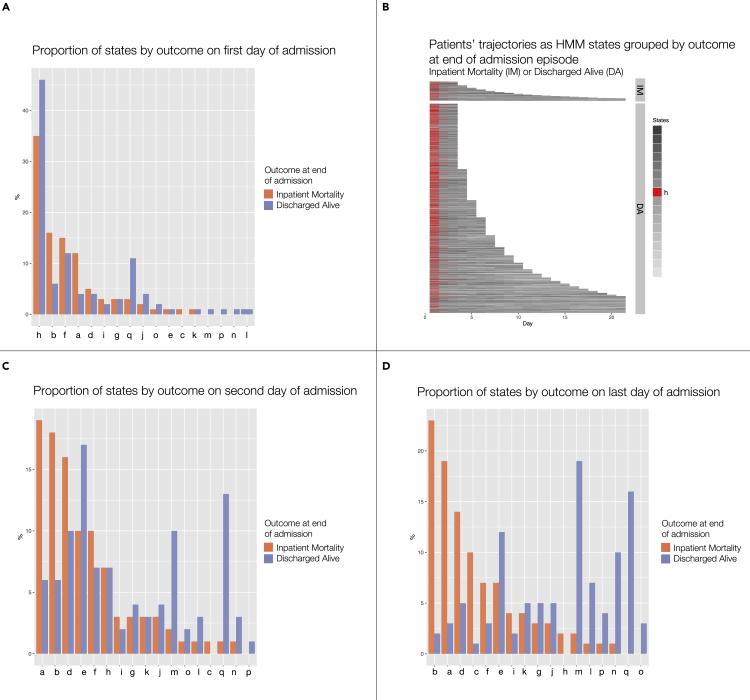
Proportion of states by day of the admission episode (A, C, and D) Relative frequency distributions of the 17 HMM states (labeled ‘a’ to ‘q’) grouped by hospital outcome (either Inpatient Mortality [IM] or Discharged Alive [DA]) on the first (A), second (C) and last (D) day of admission episodes. The most common HMM state on day 1 for all patients is ‘h’, whereas on the second and last days of admission the distribution of states differs depending on whether patients are discharged alive or die during the admission episode. (B) Representation of patients’ trajectories using the HMM states during the first 21 days of admission episodes, with AEs grouped by outcome at the end of admission. HMM state ‘h’ is highlighted in red, and this shows that state ‘h’ almost uniquely occurs on day 1 on admission episodes, whether patients die or are discharged alive.

Figure 3 shows most patients are represented by the same state, ‘h’, on day one of their AE, whether they were discharged alive or died (Figures 3A and 3B). However, on day two patients transition to a greater range of states and the distribution of these differ depending on whether patients die during the inpatient episode or not (Figure 3C). These differences are even greater when examining the last day of admission (Figure 3D), consistent with the visualization of inpatient trajectories shown in Figure 2C. Thus, without any knowledge of the final hospital outcome, the HMM has uncovered hidden states in the vital signs and blood test data that are visually associated with risk of IM throughout the AE.

Table 1 shows the visual clinical interpretation of each state using information from the HMM output. This output is exemplified in Figure 4 with the output for other states detailed in Figures S1–S16.

**Table 1:** Description of Hidden Markov Model states including key features and visual associations

	State	Key features	Key associations
**Disease-like states**			

o	Hepatic	Abnormal liver function tests	Primary diagnosis: digestive system (K) & neoplasms (C)
j	Stable renal	Abnormal renal function tests	Primary diagnosis: genitourinary system (N)
a	Unstable renal	Abnormal renal function tests	Primary diagnosis: genitourinary system (N) Clinical outcome: inpatient death
l	Stable but static renal	Abnormal renal function tests	Clinical outcome: Discharge alive
i	Blood dyscrasia	Abnormal full blood count (wide variance for most values)	Primary diagnosis: neoplasms (C) & diseases of the blood (D)
g	Bone marrow suppression	Abnormal full blood count (low values)	Primary diagnosis: neoplasms (C) & diseases of the blood (D)

**Admission-like states**			

h	Acute presentation	High WBCs, Hemoglobin, Haematocrit & abnormal vital signs	Stage of Admission: Day 1
e	Treatment response (1)	All values nearer group mean compared to Acute Presentation-like state	Stage of Admission: Day 2 to Day 8 (or discharge)
d	Treatment response (2)	Low values of some WBCs with other values around group mean	Stage of Admission: Day 2 to Day 8 (or discharge)
m	Early discharge	All values near group mean	Stage of Admission: last days of short admission episodes
n	Pre-discharge	All values near group mean except creatinine (lower), urea (lower) and platelets (higher)	Stage of admission: last days of long admission episodes

**Physiological-like states**			

b	Early inflammatory response	Markedly high WBCs, urea, and abnormal vital signs	Clinical outcome: inpatient death
k	Resolving inflammatory response	High WBCs but vital signs nearer to group mean	Clinical outcome: long length of stay
p	Autoimmune/atopic	High basophils, eosinophils and lymphocytes	Clinical outcome: Weakly associated with discharge alive
q	Acute thrombotic	High hemoglobin, hematocrit and lymphocytes	Clinical outcome: Short length of stay and discharge alive Primary diagnosis: Diseases of the circulatory system (I) and symptoms/signs/clinical findings (R)
c	Prolonged illness	Most values abnormal but especially respiratory rate (high) and hemoglobin (low)	Admission stage: more common after Day 4
f	Other illness presentation	All parameters at or near group mean	Admission stage: More common in first 3–4 days

Summary of states and their most relevant characteristics (further detail in Tables S2 and S3). WBC, white blood cells.

**Figure 4:**
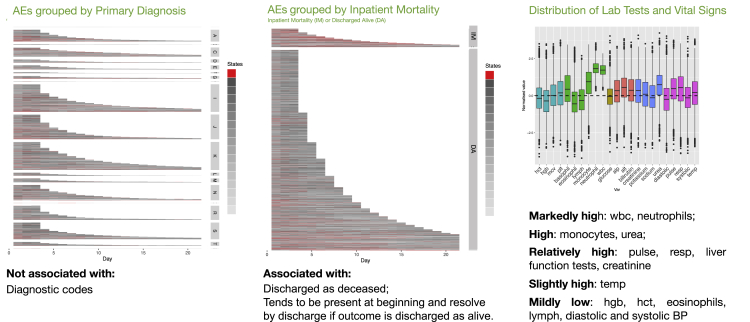
Early inflammatory response-like state Patients’ trajectories (up to 21 days) visualized as HMM states, with each patient day of each admission episode assigned one state. Admission episodes are grouped by the primary admission diagnosis as characterized by the top level International Classification of Diseases-10 code (left) and by hospital outcome at discharge (center), with *early inflammatory response-like* state highlighted in red. This visualization shows that early inflammatory response-like state occurs in admissions characterized by several different primary admission diagnoses but is more common in patients who die compared to those discharged alive. The distribution of blood test results and vital signs data for days represented as Early inflammatory response-like state by the HMM is shown on the right. On average, this state is characterized by markedly high white blood cells (neutrophils), with a high respiratory rate, pulse rate and low blood pressure. AE, admission episode; ID, inpatient death; DA, discharged alive; LOS, length of stay. HMM outputs for all other states detailed in Figures S1–S16.

Each state could be interpreted by the expert clinician, who also grouped states using an overall classification system, based on the predominant state feature: *‘Disease-like’*, *‘Admission-like’* and *‘Physiological-like’*.

*‘Disease-like’* states are characterized by over-representation of the state in patients sharing a common primary admission diagnosis and/or a pattern of blood test abnormalities reflecting dysfunction in a particular organ. For example, *‘Hepatic-like’* state is defined by abnormalities in liver function tests and over-representation of the state in AEs with ‘Neoplasms’ or ‘Diseases of The Digestive System’ as the admission diagnosis. This pattern is consistent with the liver being an organ of the digestive system and a common site for metastatic neoplasms.

The predominant feature for *‘Admission-like’* states is over-representation of the state at a particular stage of the AE, either the beginning (Day 1), middle (Day 2 onwards) or in the days leading up to discharge. These states are not over-represented in one diagnosis code but have distinct patterns with respect to the distributions of biochemical and physiological variables, and the clinical interpretation of these is consistent with the temporal distribution of the state within the AE. For example, *‘Acute Presentation-like’* state is associated with Day One. Values are higher than average for hemoglobin, hematocrit, total WBCs, and neutrophils, and all vital signs are ‘abnormal’. This pattern is consistent with higher illness acuity at the point of hospital admission and common conditions causing older patients to access emergency inpatient treatment, such as acute infections or coronary syndromes.^16^^,^^17^

*‘Physiological-like’* states are characterized by patterns of ‘abnormality’ in blood test and vital sign values, that clinicians might recognize as common physiological responses to a range of insults. For example, Figure 4 shows *‘Early Inflammatory Response-like’* state, which is characterized by high respiratory rate, heart rate, WBCs, neutrophils and urea and low blood pressure and is over-represented in patients with IM. This is similar to the clinical description of the Systemic Inflammatory Response Syndrome (SIRS).^18^

The HMM algorithm was trained again in the ‘training and validation’ dataset and assessed in the ‘hold-out test’ dataset. All states could be mapped except ‘*Other Illness Presentation-like*’ state. Means in both datasets were highly correlated (Pearson, r = 0.94), with outliers corresponding to variables of the single unmapped state (Figure S17).

### Statistical analysis of HMM states

**Figure 5:**
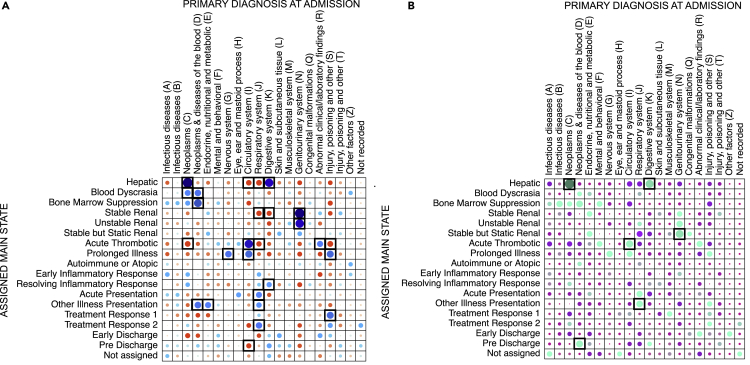
Pearson residuals plots showing associations between patients’ assigned main states and primary diagnosis at admission (A) ‘Training and validation’ dataset; (B) ‘Hold-out test’ dataset. Patients’ assigned main state was computed as the state most frequently assigned to days of their admission episode. The primary admission diagnosis is represented as the top level of the International Classification of Diseases-10 code. Blue (A) and green (B) circles indicate positive associations, with larger circles indicating stronger associations; red (A) and purple (B) circles indicate negative associations, with larger circles indicating stronger associations. Black boxes indicate statistically significant associations (p value <0.001 with Bonferroni correction). Note that information on primary admission diagnosis was not presented to the Hidden Markov Model during training.

Chi-square tests supported the visual clinical interpretation. For example, Figure 5A shows *‘Disease-like’* states exhibited strongest associations with admission diagnoses and overall, 12 States (71%) showed at least one association with a diagnosis code (p<0.001 with Bonferroni correction), with similar patterns observed in the ‘hold-out test’ dataset (Figure 5B).

Figure S18 also shows that *‘Early Inflammatory Response-like’* and ‘*Unstable Renal-like*” states were strongly associated with IM, confirming associations observed in the visual clinical interpretation, with *‘Early Inflammatory Response-like’* state also associated with PDM (Figure S3C). Other trends added to clinical interpretations. For example, *‘Prolonged Illness-like’* state generally occurred 4–5 days into an AE and was characterized by low hemoglobin and hematocrit whereas all other parameters were high, especially respiratory rate (Table 1 and Figure S15). One hypothesis, after visual clinical interpretation, was that an intervention had occurred during the AE. Consistent with this, *‘Prolonged Illness-like’* state was strongly associated with discharge by a surgical specialty (Figure S18).

Associations did not always add to the clinical interpretation. For example*, ‘Treatment Response-like 1 and 2’* and ‘*Other Illness Presentation-like’* states, which were not as confidently classified in the visual clinical interpretation, remained less clearly defined than other states.

### Prediction models

Logistic Regression (LR) and Random Forest (RF) were trained on the prediction of clinical outcomes, with predictor variables including patients’ representations during the first three days of admission (either MVTS data or HMM state spaces) and other phenotypic variables. Across all models, Table 2 shows that higher performances on ROC-AUC and weighted ROC-AUC were observed for models trained with MVTS compared to states representations, although the HMM states performances were comparable. Almost all models showed highest performance when trained with either Day 3 (D3) or Days 1–3 (D1D2D3) input variables. The inclusion of phenotypic information modestly improved the performance for IM and clinical outcome at 30-day post-discharge.

**Table 2:** Performance of predictive models

Predicted outcome	Datarepresentation	Algorithm	Input data		ROC-AUC	
			Days	Phenotypic information	Training dataset	Test dataset
Inpatient Mortality	MVTS	RF	D3	YES	0.85	0.86
	STATES	RF	D3	YES	0.79	0.63
Clinical outcome	MVTS	RF	D1D2D3	YES	0.68	0.68
	STATES	RF	D3	YES	0.67	0.58
Diagnosis at admission	MVTS	RF	D1D2D3	–	0.76	0.75
	STATES	RF	D1D2D3	–	0.65	0.54
Diagnosis at discharge	MVTS	RF	D1D2D3	–	0.65	0.75
	STATES	RF	D2D3/D1D2D3^a^	–	0.6	0.54

ROC-AUC (and weighted ROC-AUC) results for the models with highest performance on the prediction of inpatient mortality, 30-day clinical outcome, diagnosis at admission and diagnoses at discharge. RF, Random Forest; MVTS, Multivariate time series; Phenotypic information includes age, sex, Clinical Frailty Scale score, and admission diagnosis.

a Same performance with both input datasets.

Prediction of IM using RF and D3 MVTS predictor variables was the best performing model overall (Tables 2 and S6–S9). The top five features for this model were respiratory rate, eosinophil count, urea, lymphocytes and neutrophils. These closely resemble features characterizing the *‘Early Inflammatory Response-like’* state generated by the HMM (Figure 4), itself associated with inpatient mortality.

Similar weighted ROC-AUC performances were achieved for prediction of clinical outcome at 30 days with the highest obtained by the RF model trained with MVTS data on D1D2D3 and phenotypic information (Tables S10–S14). The averaged results were mainly driven by the performance achieved on the prediction of the majority class (DA). Results for inpatient death did not differ considerably from those previously observed in the binary classification of this outcome but performance for other outcome classes was limited, mostly because of small numbers of outcome events, especially for PDM and PDRM.

The predictions of primary diagnosis at admission (PDA) and discharge (PDD) are overall lower than for other outcomes and differ by diagnosis code, usually being higher for those with more instances in the dataset (Tables S15–S18).

## Discussion

We demonstrate clinical time-series numerical data, routinely recorded during older adult AEs, can be processed into a research ready ML dataset. Furthermore, we prove the ability of an unsupervized ML technique, HMM, to extract clinically interpretable signals from this data and show that ML model outputs can be transparently evaluated, helping clinicians understand ML models. Imputed observations did not influence results and all 17 HMM state spaces could be retrospectively interrogated and interpreted by an expert clinician. These interpretations were supported by formal association analyses between states and hold-out clinical information, such as diagnoses and final hospital outcome, suggesting real biological signals were captured. Accordingly, visualization of AEs using the HMM states representations proved informative, clearly showing older adult patients whose condition was moving toward a stable ‘*discharge-like*’ state as their AE progressed versus those remaining ‘unstable’, at ongoing risk of mortality.

The strikingly different state distributions by IM deserve further consideration. Although ‘*Acute Presentation-like*’ state is almost uniquely observed on the first day for all patients, consistent with a common need to stabilize acute illness, patients transitioned to a greater range of states from Day 2 onwards depending on their hospital outcome. This highlights how careful ML modeling of daily, or finer grain, vital signs and blood tests information could be used to better inform clinicians of patient risk across the entirety of an AE. Current decision support tools aimed at identifying deteriorating patients have not usually been developed considering temporal trends in physiological status, and employ simple risk factor categorizations for scoring at the bedside.^19^ However, the implementation of hospital EHRs offers an opportunity to develop new tools capable of considering more comprehensive and complex clinical information. For example, tools that can utilize continuous risk factor estimates^20^ and consider the temporal sequence of data,^12^ perhaps focusing on days two and three of AE as patients’ clinical trajectories diverge.

It was also interesting to observe that the HMM states appeared to coalesce into one of three higher level classifications, which made biological and clinical sense. The conceptual ‘state’ of a hospitalized patient at any moment will depend on their disease burden (‘*Disease-like*’), their physiological response to this burden (‘*Physiological-like*’) and their response to medical intervention (‘*Admission-like*’). This not only reveals insights into how ML models establish patterns within large, complex clinical datasets, enabling healthcare practitioners to better understand ML models, but these findings have potential clinical applications. For example, *‘admission-like’* states characterize the temporal evolution of AEs from presentation to treatment and discharge states. Such modeling could be further developed for purposes such as the ‘forecasting’ of bed capacity, helping hospital managers prioritize resources, or development of tools to support identification of patients clinically fit for discharge. These tools could facilitate early discharge planning^21^ and use of early supported discharge pathways.^22^ Older adults are frequent users of inpatient services and even relatively modest gains in healthcare delivery and effectiveness could have large system wide effects.^23^

Following on from this, the discriminative learning models performed reasonably well, especially given that we focused on a limited number of commonly measured predictor variables and limited our cohort to those with an AE of ≥3 days. This excluded the most acutely unwell and fittest patients, whose outcome was perhaps easiest to predict. Higher prediction performance was achieved with the MVTS as input data compared to HMM states, reflecting the reduction of information from a multivariate to univariate time-series. However, models employing states representations still performed comparably well, validating the HMM approach. Furthermore, use of both techniques (generative HMM and discriminative modeling with LR and RF) provided additional opportunities to understand and learn from ML outputs. For example, both identified states and features representative of a strong inflammatory response as important for mortality, emphasising the importance of prompt identification and treatment of SIRS, and the conditions causing it, in clinical practice.^18^

The increasing availability of large scale EHR data has incentivized research on time-based modeling of clinical data, with HMM as the selected approach to model disease stages^13^^,^^15^^,^^24^^,^^25^^,^^26^^,^^27^ or predict clinical outcomes.^28^^,^^29^^,^^30^ Gupta et al., describe a methodological approach similar to the one presented here, with data pre-processing and imputation of missing observations and training of a 2-state HMM using three input vital signs variables. In contrast, our work attempts to train a disease-agnostic HMM without a clinically predefined number of states and with a large number of heterogeneous laboratory tests and vital signs.

In summary, we generated an ML research ready dataset using hospital EHR data and extracted clinically interpretable signals, providing an informative view of older adult inpatient trajectories. This generates hypotheses around potential future applications of traditional and advanced ML methods in the development of clinical decision support tools for EHR systems, which should be explored in future work.

### Limitations of the study

Our dataset has some limitations. It was extracted from a single hospital with the final cohort subject to strict inclusion criteria, aiming to regularize the dataset whilst limiting missingness. Future work with less restrictive or shorter time-bins might explore the best way to include patients and information excluded here,^31^ using approaches such as deep learning^32^ or exploring alternatives to imputation.^7^ The clinical interpretations and classifications provided for the HMM states also require careful consideration. We acknowledge that insisting every patient day is assigned a single state is a crude approximation to clinical reality. Nevertheless, it was reassuring the HMM states mapped well between ‘training and validation’ and ‘hold-out test’ datasets, except for a single state. One poorly defined heterogeneous state per iteration is expected, given the HMM assigns a single state to every time point and patient, with a limited set of states.

## STAR★Methods

### Key resources table

**Table:** 

REAGENT or RESOURCE	SOURCE	IDENTIFIER
**Deposited data**		

Anonymized patient data	Cambridge University Hospitals (CUH) and EMBL-EBI	N/A

**Software and algorithms**		

R: A language and environment for statistical computing	R Core Team,^33^	https://www.R-project.org/; RRID: SCR_001905
mice (R package)	Van Buuren and Groothuis-Oudshoorn,^34^	https://CRAN.R-project.org/package=mice
imputeTS (R package)	Moritz and Bartz-Beielstein,^35^	https://cran.r-project.org/web/packages/imputeTS/
VIM (R package)	Kowarik et al.^36^	https://cran.r-project.org/web/packages/VIM/
Python software version 2.7	Python Software Foundation	https://www.python.org; RRID: SCR_008394
Python software package: hmmlearn	https://hmmlearn.readthedocs.io/en/latest/index.html	https://hmmlearn.readthedocs.io/en/latest/index.html

### Resource availability

#### Lead contact

Further information and requests for data access should be directed to and will be fulfilled by the lead contact, Victoria L Keevil (vlk20@cam.ac.uk).

#### Materials availability

No materials were used or generated in this study.

### Experimental model and subject details

Data from 61,513 patients >65 yearsold admitted as an emergency to an NHS University hospital between January 2015 and December 2019 were retrospectively retrieved from the hospital’s EHR system and de-identified by the Clinical Informatics team. The final selected cohort comprised 11,158 individuals (Table S1). Data included demographics, static clinical variables (clinical frailty scale [CFS] score,^37^ diagnoses at admission and discharge), blood test results (commonly requested tests), bedside vital signs, and hospital outcomes: inpatient mortality (IM), 30-day post-discharge readmission (PDR), and 30-day post-discharge mortality (PDM) (Table S4).

The project was approved by the NHS Health Research Authority (IRAS: 253,457), North East – Newcastle & North Tyneside 1 Research Ethics Committee (REC) (REC ref. 19/NE/0013) and by the EMBL Scientific Advisory Committee (BIAC).

### Method details

#### Study design and patient cohort

Only patients’ first AEs during the study period, with a minimum length of stay of three days, were included. The final dataset comprised 11,158 unique AEs, corresponding to the same number of unique patients, and was randomly divided into ‘training and validation’ (80% AE) and ‘hold-out test’ (20% AE) sets for exploratory and confirmatory analyses.

Phenotypic information includes age, sex, Clinical Frailty Scale score, and admission diagnosis. ‘*Rich information*’ days were defined as days with information for >14 blood tests and >4 vital signs. All other days were defined as ‘*Poor Information*’ days.

#### Pre-processing for MVTS data

23 variables denoted blood test (17/23) and vital sign (5/23) values which were normalised using inverse rank normalization using the R package.^33^ We defined 24-h bins and selected a unique observation for each variable in each bin. Blood tests are not often measured more than once per day but if multiple observations were recorded on the same day, the earliest record was selected as the unique value and the closest to these measurements for vital signs, since these are usually measured several times per day.

Daily bins were defined as ‘*rich-information*’ or ‘*poor-information*’ days depending on the number of missing observations. A ‘*rich-information*’ day was defined as a day with information for at least four vital signs and 14 blood tests, the smallest fraction of missingness leading to a sample size of around 10,000 patients. The first and last days of included AEs had to be ‘*rich-information*’ days with <⅓ of the AE length comprising ‘*poor-information*’ days.

#### Imputation of missing values

Figure S19 shows the two-step process adopted for imputation of missing data: multiple imputation (MI)^38^ of ‘*rich-information*’ days and linear interpolation (LI) imputation of ‘*poor-information*’ days. LI imputation relies on non-missing values from adjacent observations to compute the missing value in the time series^39^ and is independent of other variables and patients while MI with Predictive Mean Matching (PMM) was conducted using information from the same and other patients. Explanatory blood test and vital sign variables for each predicted variable excluded those that were highly correlated, those with the largest fractions of missingness or highly correlated missingness patterns. Age, sex, discharge specialty and primary diagnosis at admission were also included as predictors (Figure S20). The dataset was imputed ten times. We used the R^33^ packages *mice*^34^ and *imputeTS*.^35^^,^^36^

#### Generative models for multivariate time-series representation: HMM

An unsupervised HMM with Gaussian emissions, full covariance matrix and 17 states (the only defined parameter) was trained using expectation maximisation with only the MVTS as input data (Python package *hmmlearn)*. No diagnosis or outcome data was presented to the HMM and the number of states was selected in a 2-fold cross-validation process where the ‘training and validation’ set was randomly divided into two subsets, which were separately used to train a model and then fit the entire dataset. The output of both models was compared on a concordance matrix of co-occurrences for patient and day with the aim to find an association between states in both output datasets (i.e., a model trained with n states in two different datasets would lead to similar results or predicted states), while selecting a number of states large enough to capture the heterogeneity of the data.

The HMM technique can be considered as a dimensionality reduction of the multivariate space of patients with a natural time dependence that transforms MVTS data into a simpler state space maximising the likelihood of the observed data and capturing dominant aspects of the patient’s condition on each day. Inpatients’ trajectories are then represented as a univariate discrete time series, with each AE day of each patient represented as a single unique state space, instead of 23 numeric variables.

#### Prediction models

Two well established fixed frame ML techniques, Logistic Regression (LR) and Random Forest (RF), were trained on the prediction of clinical outcomes, primarily IM (i.e., patients who died in hospital at the end of AE vs those who were discharged), following registration of our hypotheses in the Open Science Framework (https://osf.io/6zp3d; Table S5). Predictor variables included patients’ representations during the first three days of admission (either MVTS data or HMM state spaces), age, sex, CFS score and admission diagnosis. LR models were trained for binary classification (IM) and RF models for binary, multiclass (prediction of five disjoint outcomes: inpatient death [ID], discharged alive [DA], PDR, PDM and post-discharge readmission and mortality [PDRM]; and prediction of primary diagnosis at admission [PDA]) and multilabel classification (prediction of diagnoses at discharge [DD]). The models were trained with the python library *scikit-learn*.

### Quantification and statistical analysis

#### Statistical analysis

Data was imputed for 25.5% of blood test values (7.8% MI; 17.7% LI) and 0.3% (0.26% MI; 0.04% LI) of vital sign values in the ‘training and validation’ dataset. There was no evidence that imputation influenced results, including HMM outputs (Figure S21 and detailed elsewhere^40^).

A clinician first inspected the distribution (mean values and variance) of vital sign and blood test values^41^ by HMM state and visually evaluated: 1) the temporal distribution of the state within AEs; 2) the distribution of the state across AEs organised by admission diagnosis; and 3) the distribution of the state by IM. Formal association analyses using Chi-square tests were conducted in R to confirm the expert interpretation (p value <0.001 with Bonferroni correction as shown in Figure 5).

#### Methods and results validation

Reproducibility of the results was assessed by re-training the HMM algorithm in the ‘training and validation’ dataset and fitting the model to the ‘hold-out test’ dataset. Variable distributions were compared and Pearson correlations between their means calculated. Chi-square tests were similarly conducted in this dataset.

Prediction performance was assessed using the area under the receiver operating characteristic curve (ROC-AUC). The ‘training and validation’ dataset was divided into a training set (80%) for 5-fold cross validation hyperparameter tuning (LR: penalty term; RF: number of trees, number of random features to sample at each split point, maximum depth of the tree, minimum number of samples required to split a node, minimum number of samples required at each leaf node, method of selecting samples for training each tree i.e., bootstrapping) and a test set (20%) for performance evaluation with the selected best parameters. The process was repeated ten times. All the trained models were then run against the test dataset without additional training. Here we describe only the performance achieved by the best models as identified in the training dataset. Detailed results of performance metrics in both datasets are reported in Tables S6–S18 and elsewhere.^40^

#### Role of the funding source

This research was supported by the NIHR Cambridge Biomedical Research Center (BRC-1215-20014). The views expressed are those of the authors and not necessarily those of the NIHR or the Department of Health and Social Care. VLK was funded by an MRC/NIHR Clinical Academic Research Partnership Grant (CARP; grant code: MR/T023902/1). VT is supported by Cancer Research UK. EB and TF were funded by the EMBL European Bioinformatics Institute (EMBL-EBI). The funders had no role in developing the research question or the study protocol.

## Data Availability

• The anonymised data reported in this study cannot be deposited in a public repository because were used under license for the current study, and so are not publicly available. To request access, contact the lead author Victoria L Keevil from Cambridge University Hospitals NHS Foundation Trust. Reasonable data requests will be considered by the authors with permission of Cambridge University Hospitals NHS Foundation Trust. Requests for data access should be directed to the lead contact. • All original code has been deposited on GitHub (GitHub: https://github.com/mariaheza/hmm_inpatient_trajectories) and is publicly available as of the date of publication. DOIs are listed in the key resources table. • Any additional information required to reanalyse the data reported in this paper is available from the lead contact upon request.
