# Supplementary material for: Using machine learning to model older adult inpatient trajectories from electronic health records data
Source: iScience. 2022 Dec 24;26(1):105876. doi: 10.1016/j.isci.2022.105876 (PMC9860485; doi:10.1016/j.isci.2022.105876)
Supplement: Document S1. Figures S1–S21 and Table S1–S18 [file mmc1.pdf]

iScience, Volume *26*

Supplemental information

Using machine learning to model older adult inpatient trajectories from electronic health records data

Maria Herrero-Zazo, Tomas Fitzgerald, Vince Taylor, Helen Street, Afzal N. Chaudhry, John R. Bradley, Ewan Birney, and Victoria L. Keevil

Figure S1: Visual interpretation of *Hepatic-like* state. Related to Figure 4.

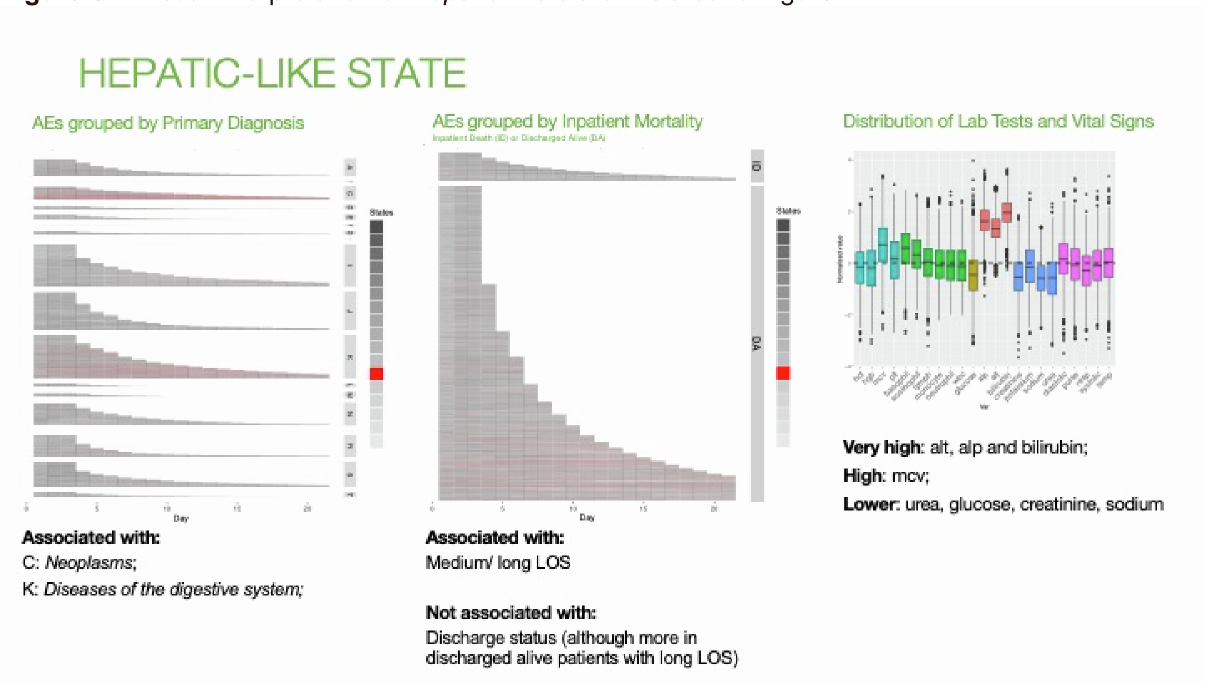

Figure S2: Visual interpretation of *Stable renal-like* state. Related to Figure 4.

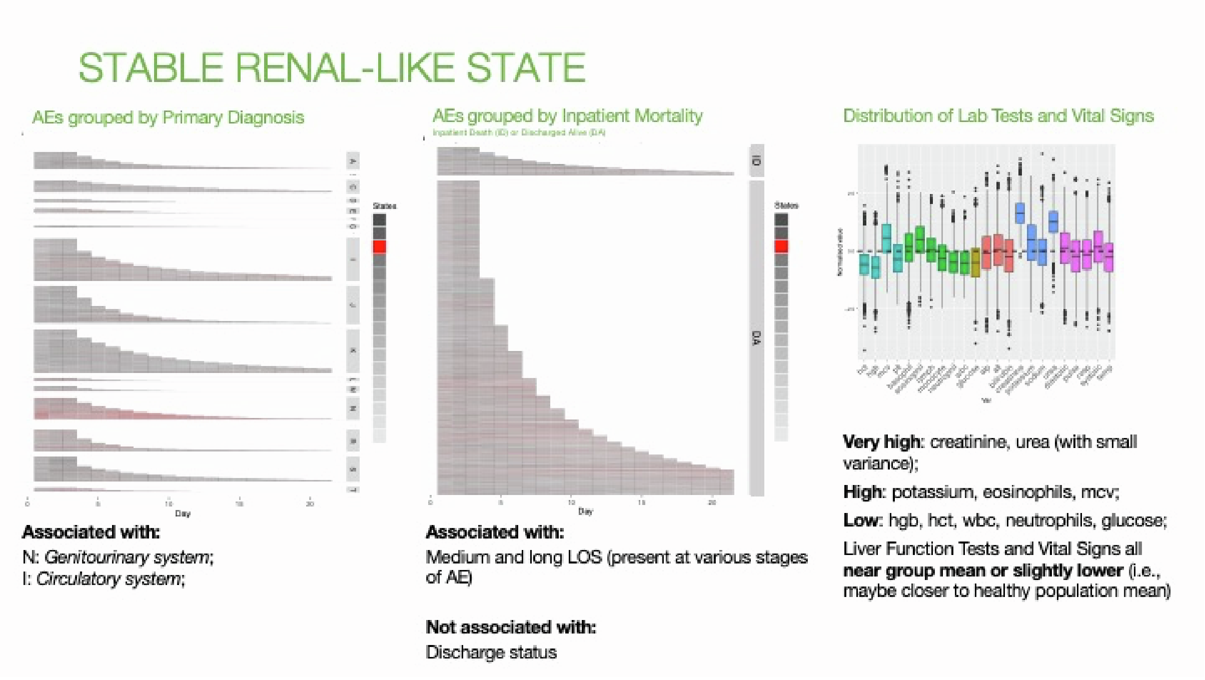

Figure S3: Visual interpretation of *Unstable renal-like* state. Related to Figure 4.

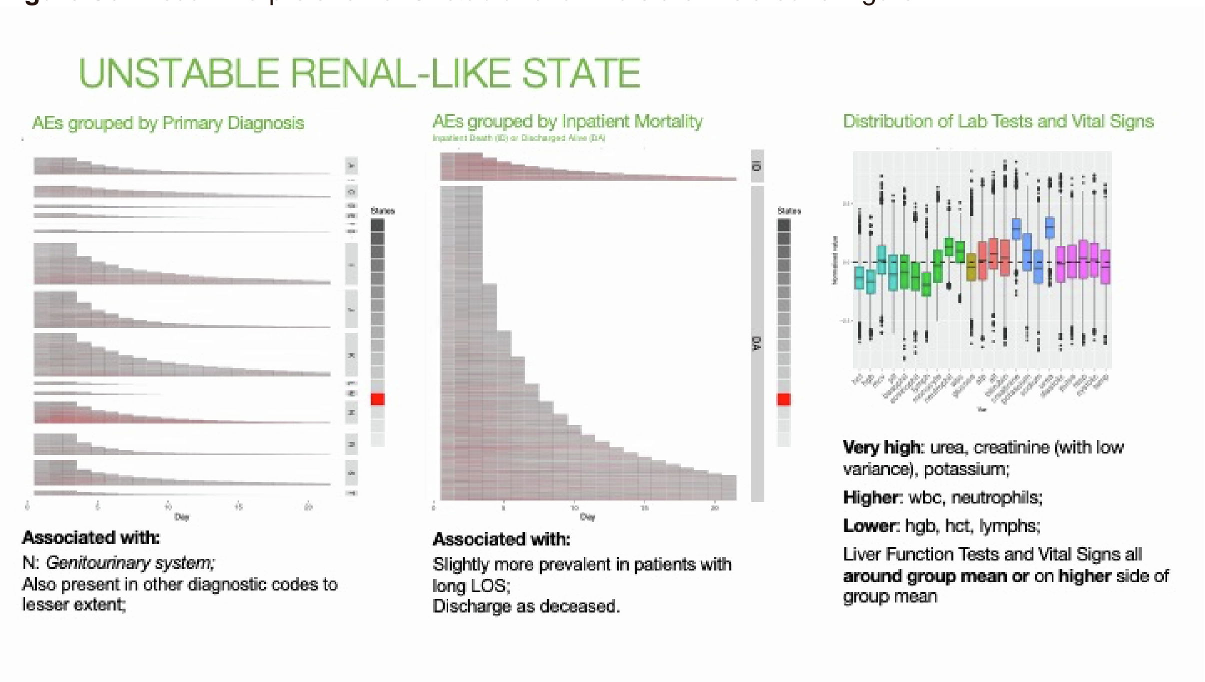

Figure S4: Visual interpretation of *Static renal-like* state. Related to Figure 4.

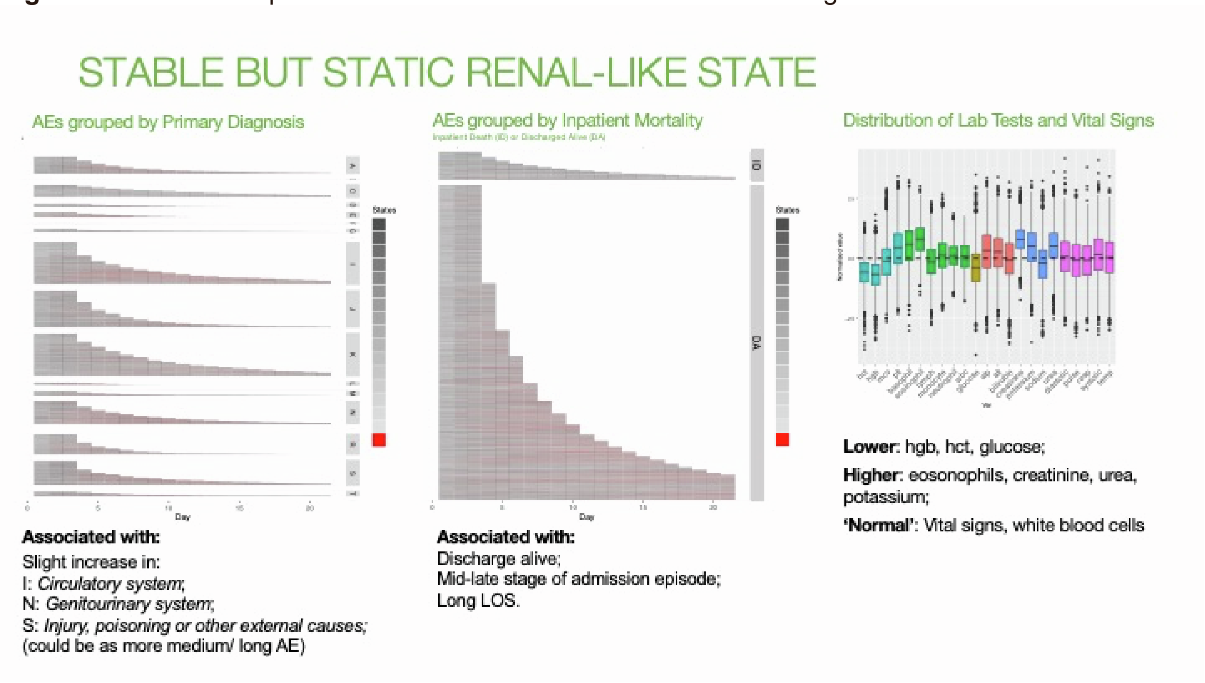

Figure S5: Visual interpretation of *Blood dyscrasia-like* state. Related to Figure 4.

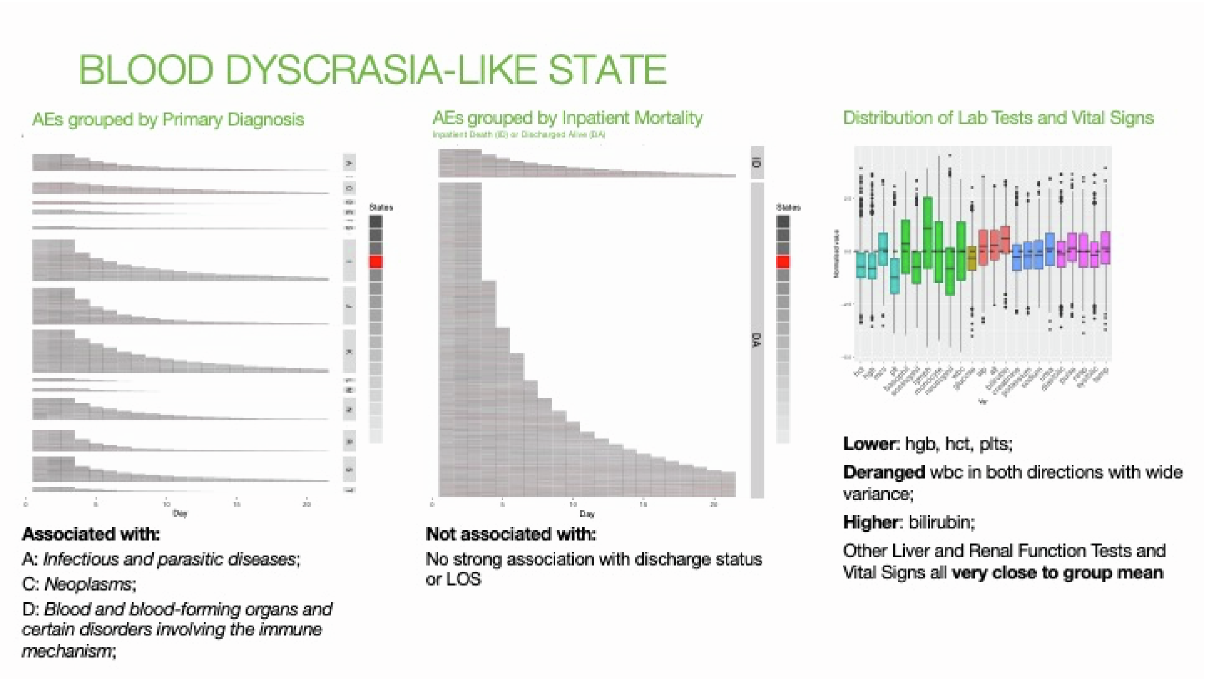

Figure S6: Visual interpretation of *Bone marrow suppression-like* state. Related to Figure 4.

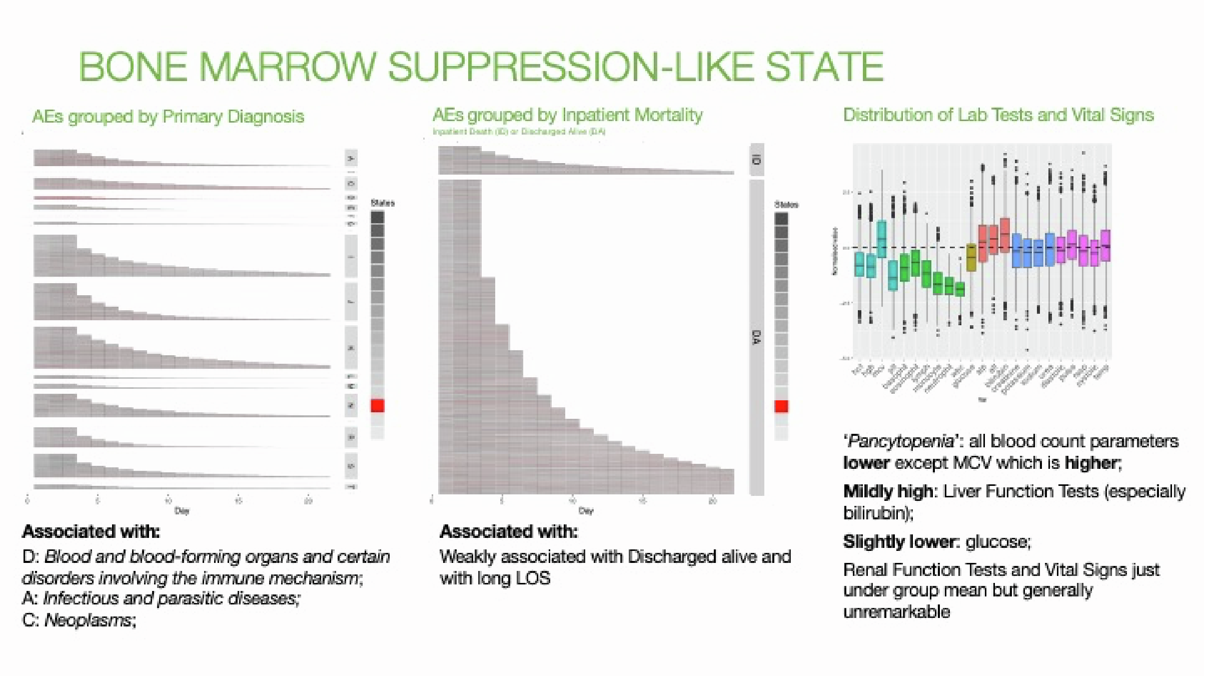

Figure S7: Visual interpretation of *Acute presentation-like* state. Related to Figure 4.

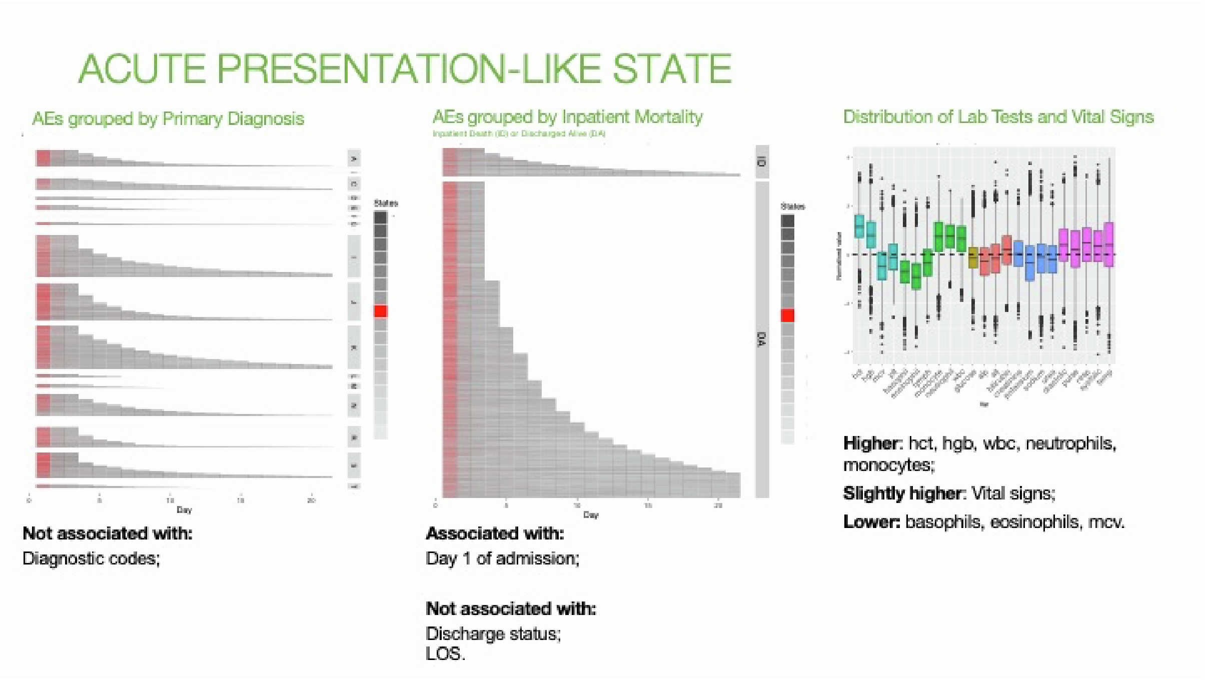

Figure S8: Visual interpretation of *Treatment response-like* state (1). Related to Figure 4.

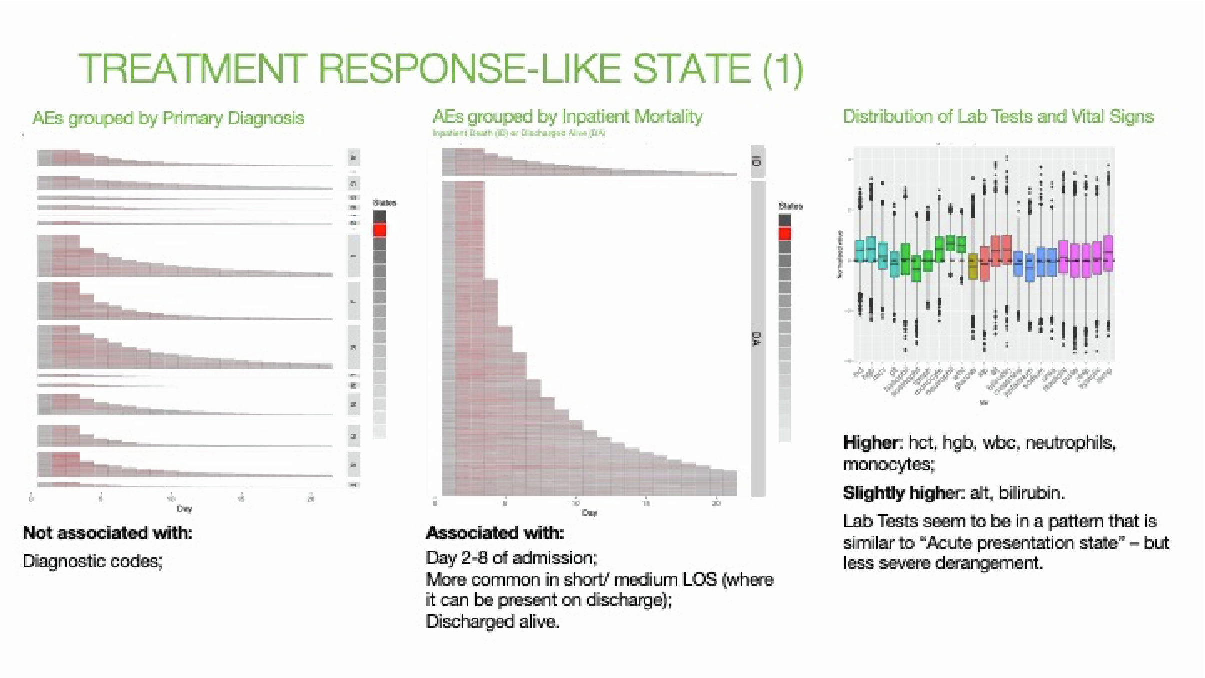

Figure S9: Visual interpretation of *Treatment response-like* state (2). Related to Figure 4.

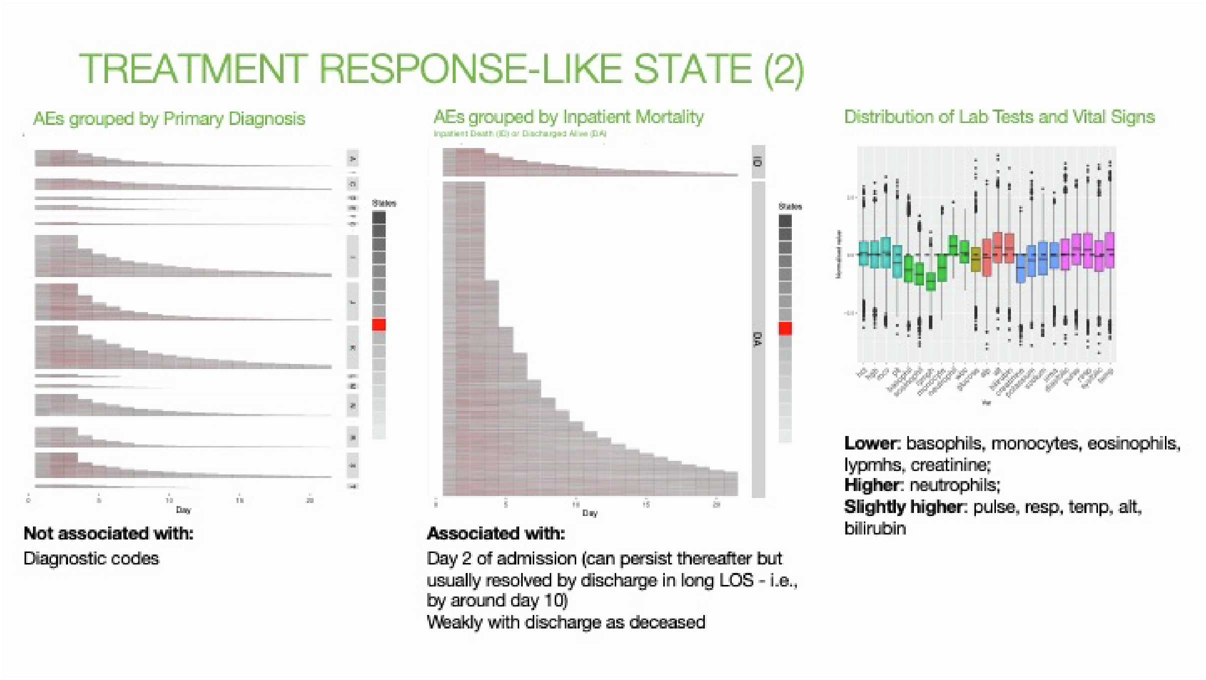

Figure S10: Visual interpretation of *Early discharge-like* state. Related to Figure 4.

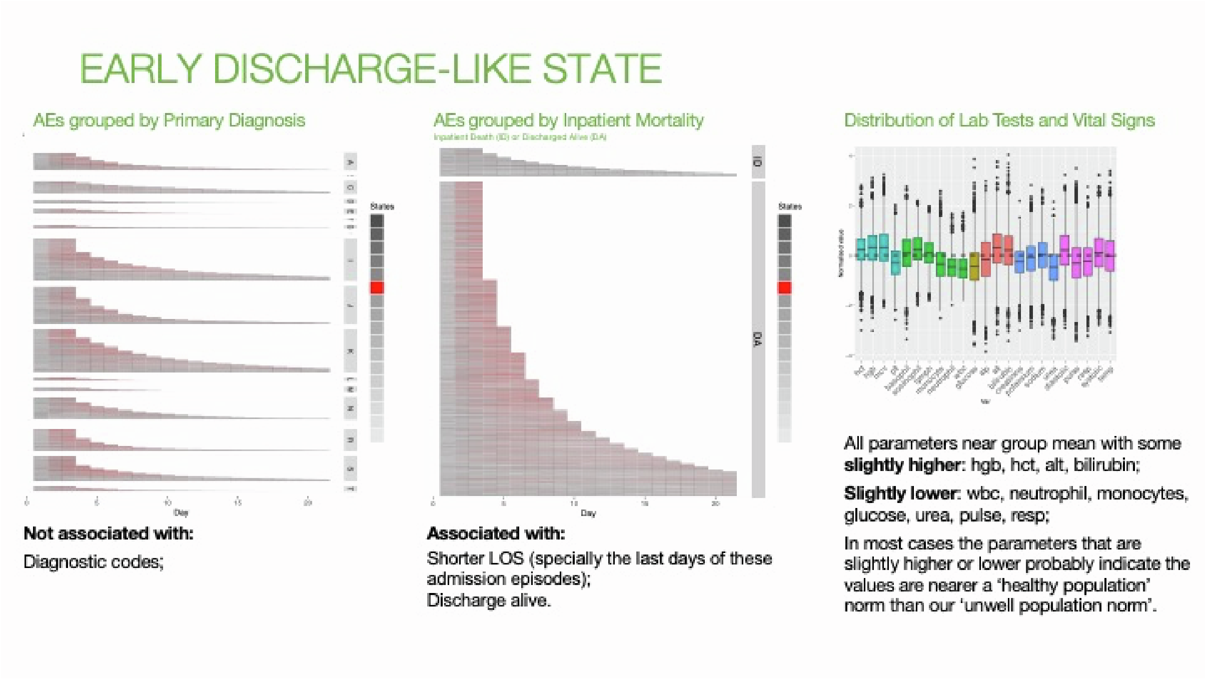

Figure S11: Visual interpretation of *Pre-discharge-like* state. Related to Figure 4.

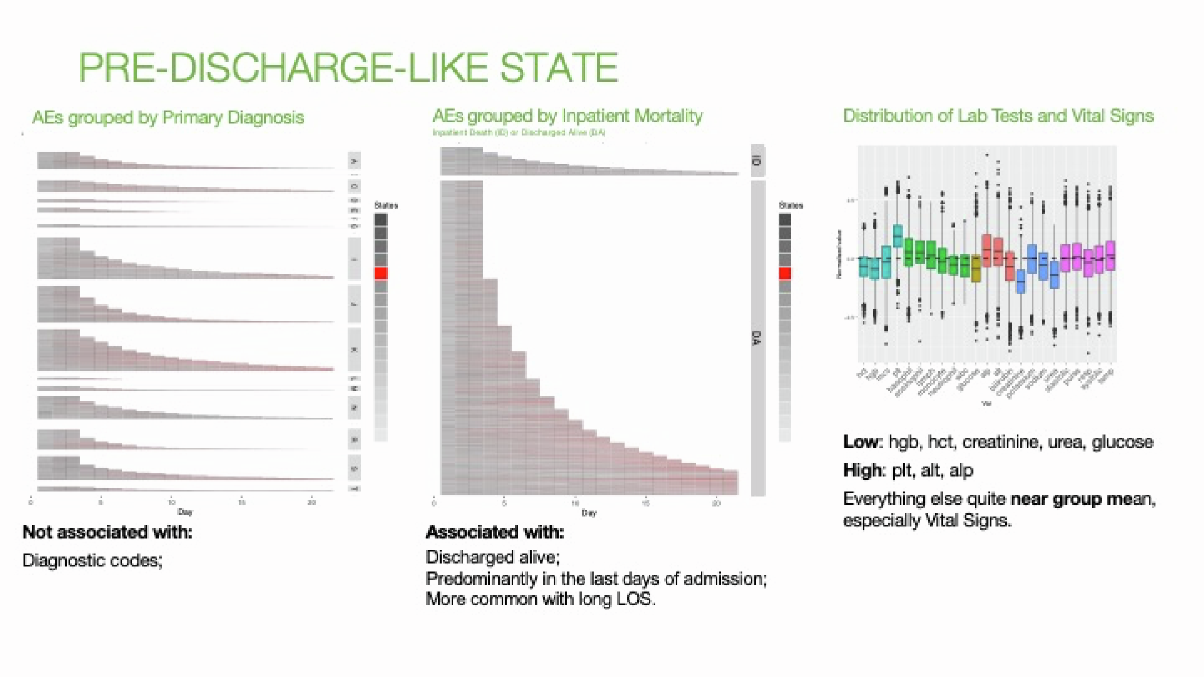

Figure S12: Visual interpretation of *Resolving inflammatory response-like* state. Related to Figure 4.

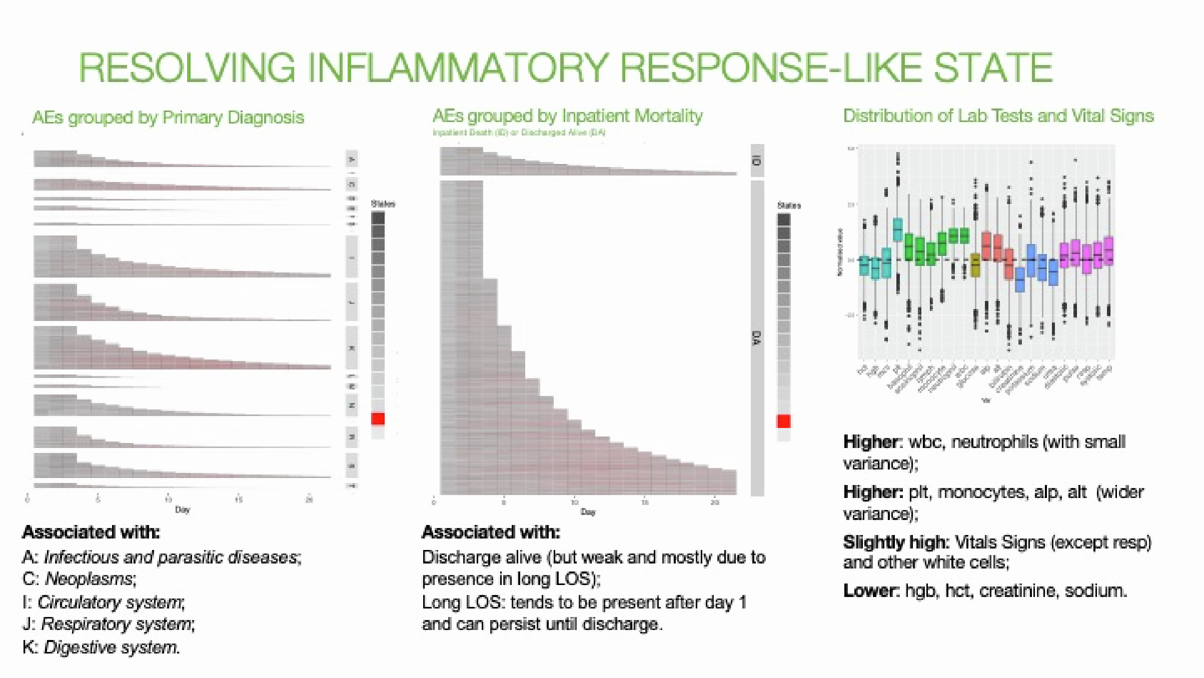

Figure S13: Visual interpretation of *Autoimmune/atopic-like* state. Related to Figure 4.

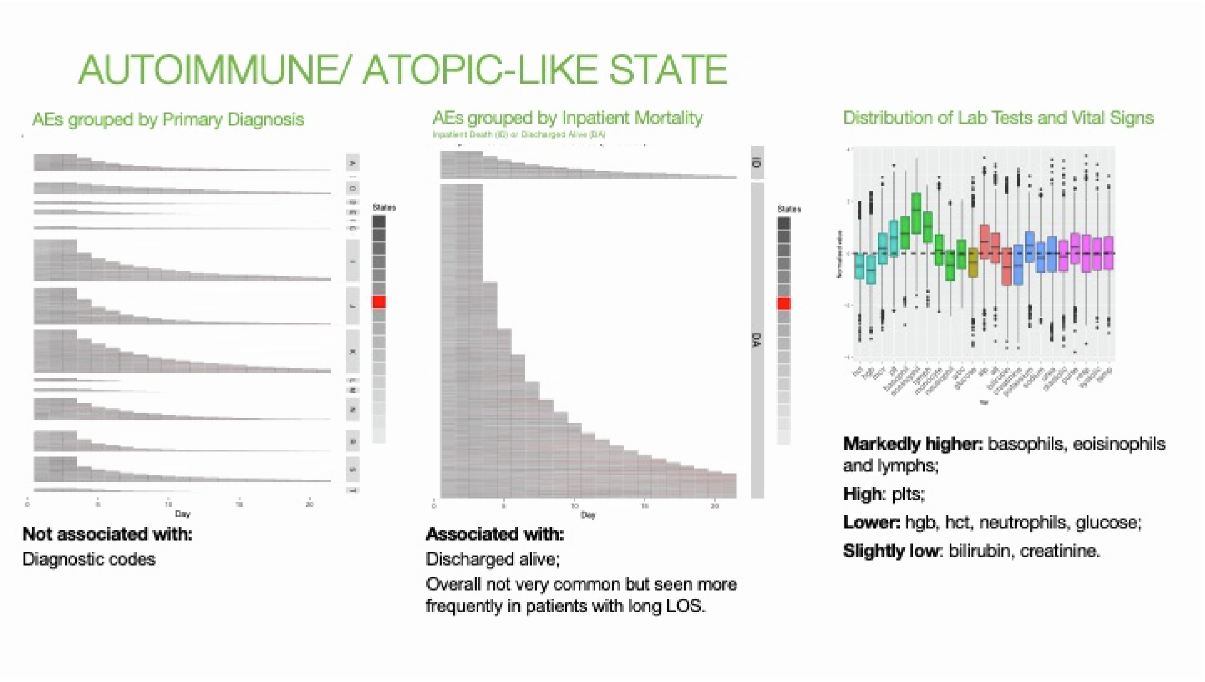

Figure S14: Visual interpretation of *Acute thrombotic-like* state. Related to Figure 4.

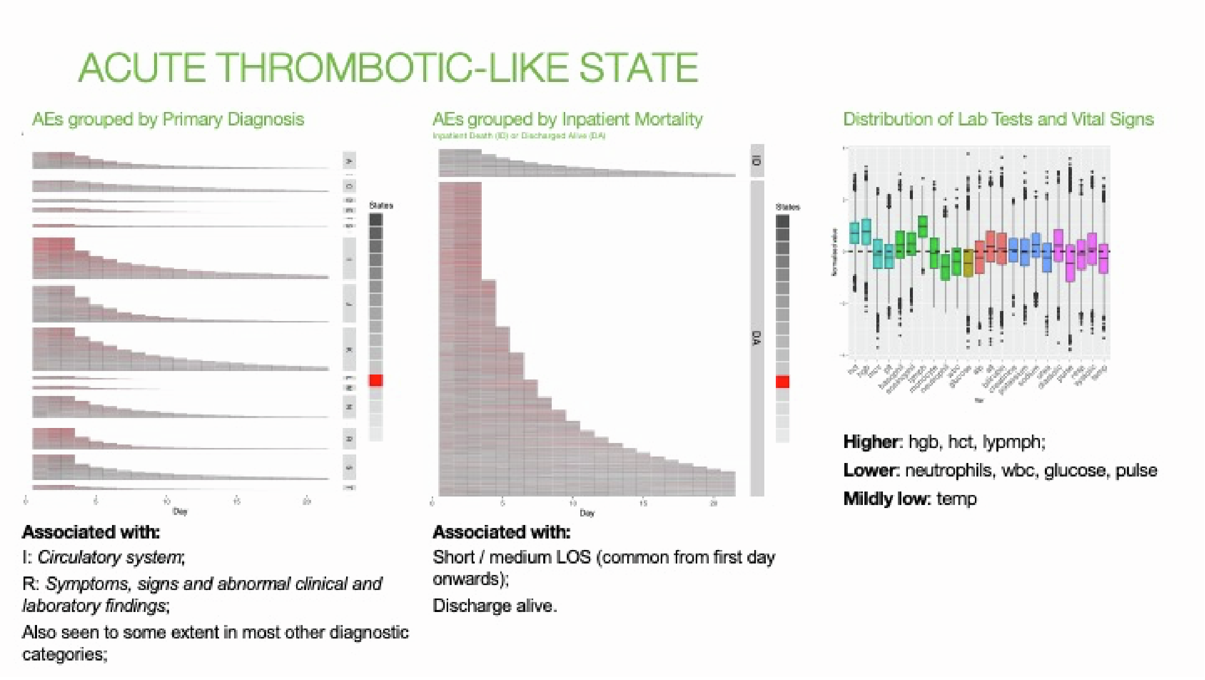

Figure S15: Visual interpretation of *Prolonged illness-like* state. Related to Figure 4.

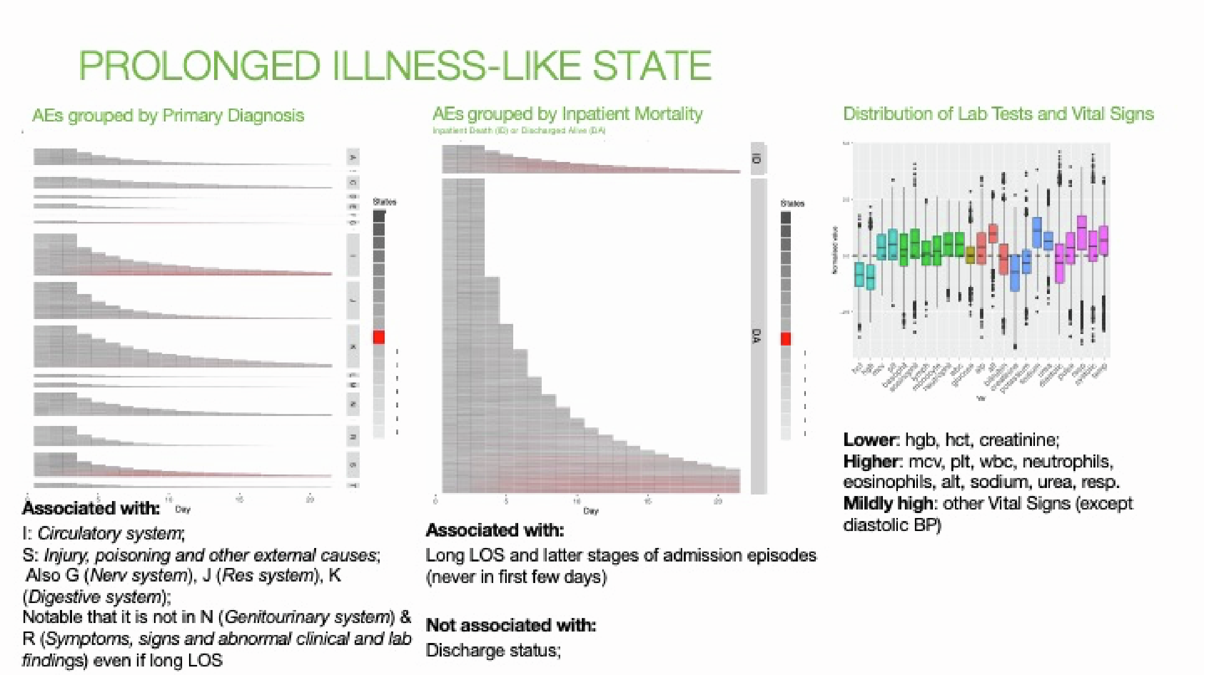

Figure S16: Visual interpretation of *Other illness-like* state. Related to Figure 4.

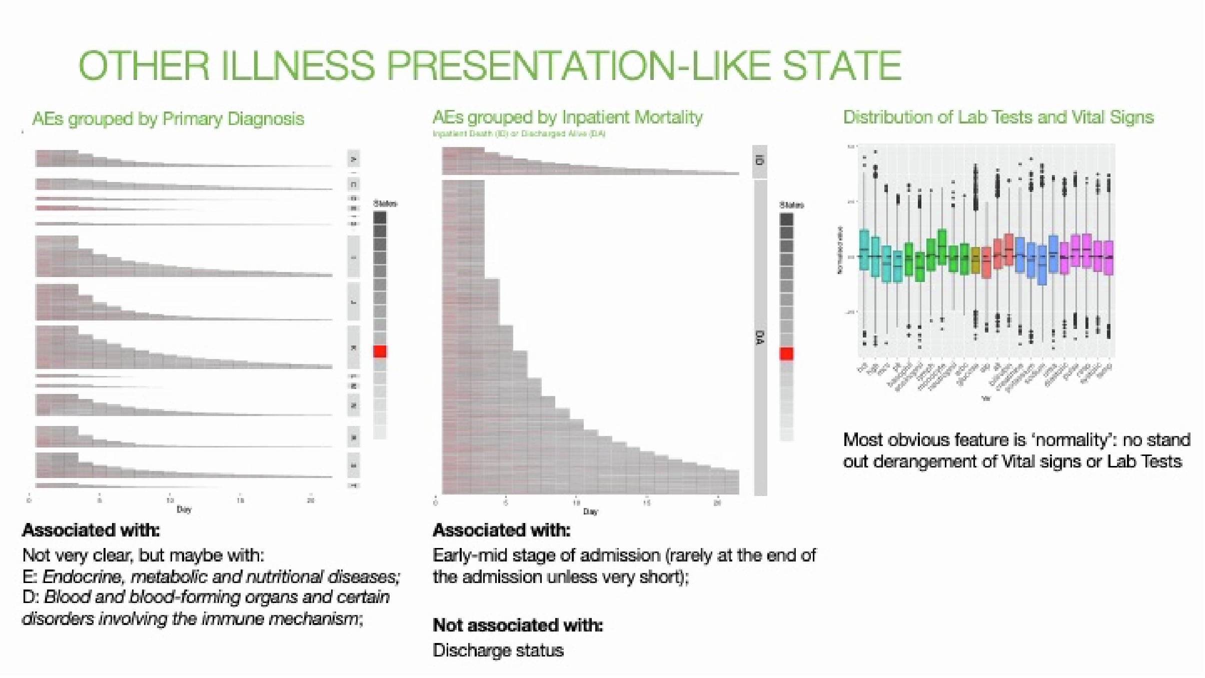

Figure S17: Correlation between mean values of laboratory test results and vital signs variables in the ‘training and validation’ and ‘hold-out test’ datasets. Related to Figure 5 and STAR Methods.

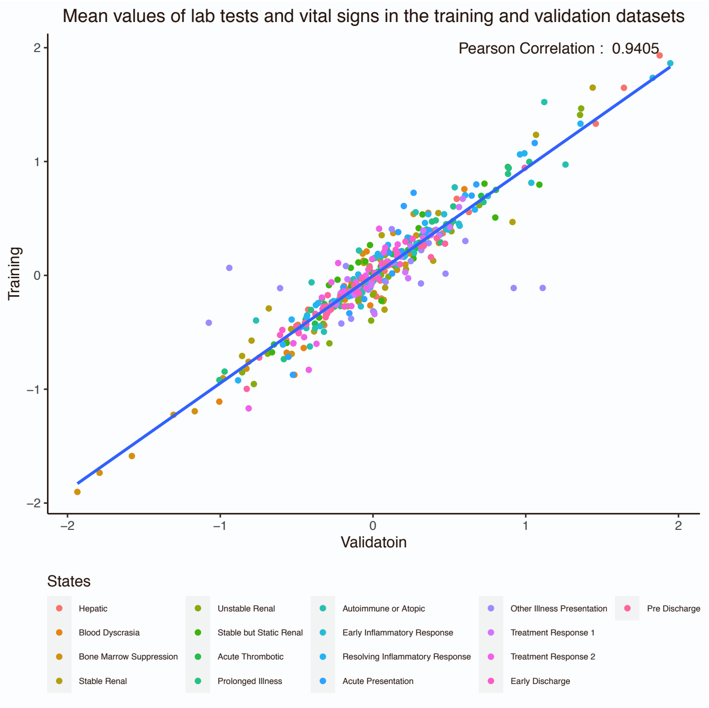

Figure S18: Pearson residuals plots showing associations between patients' assigned main states and clinical information not presented to the Hidden Markov Model. Related to Figure 5.

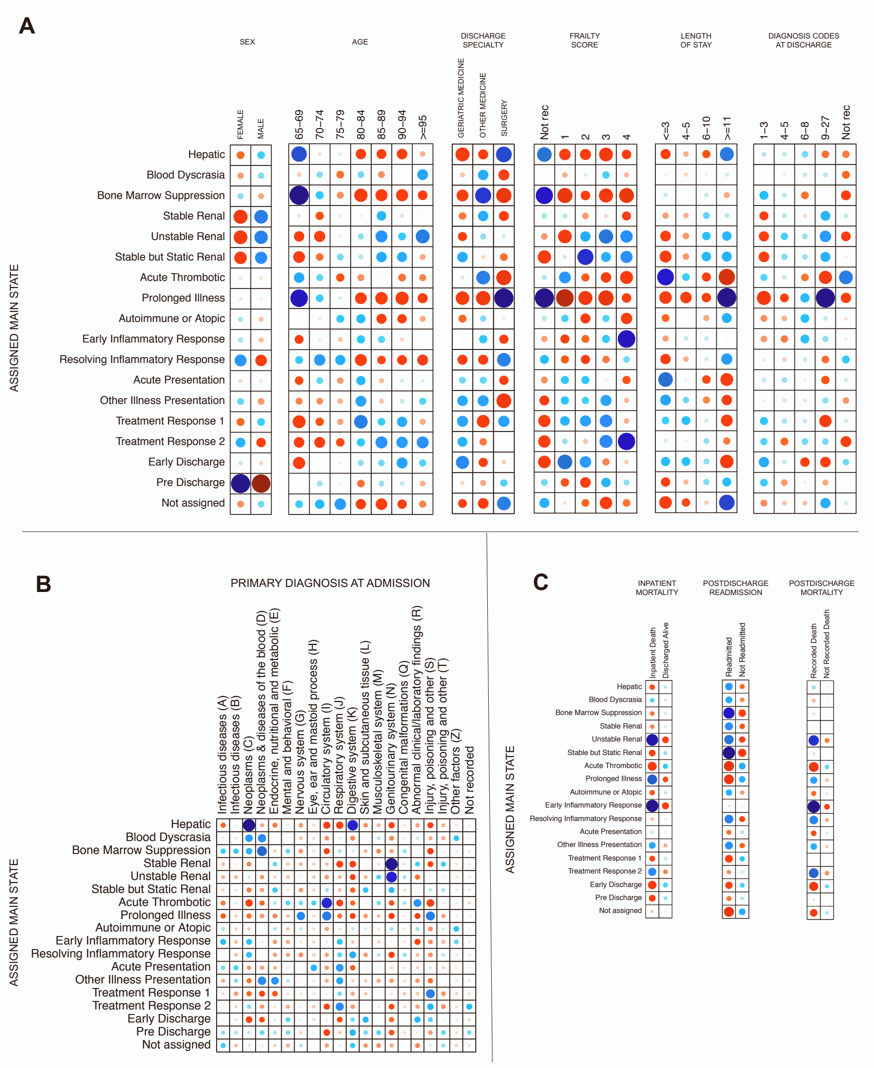

Blue: positive associations; red: negative associations.

Figure S19: Summary of data pre-processing and imputation**.** Related to STAR Methods.

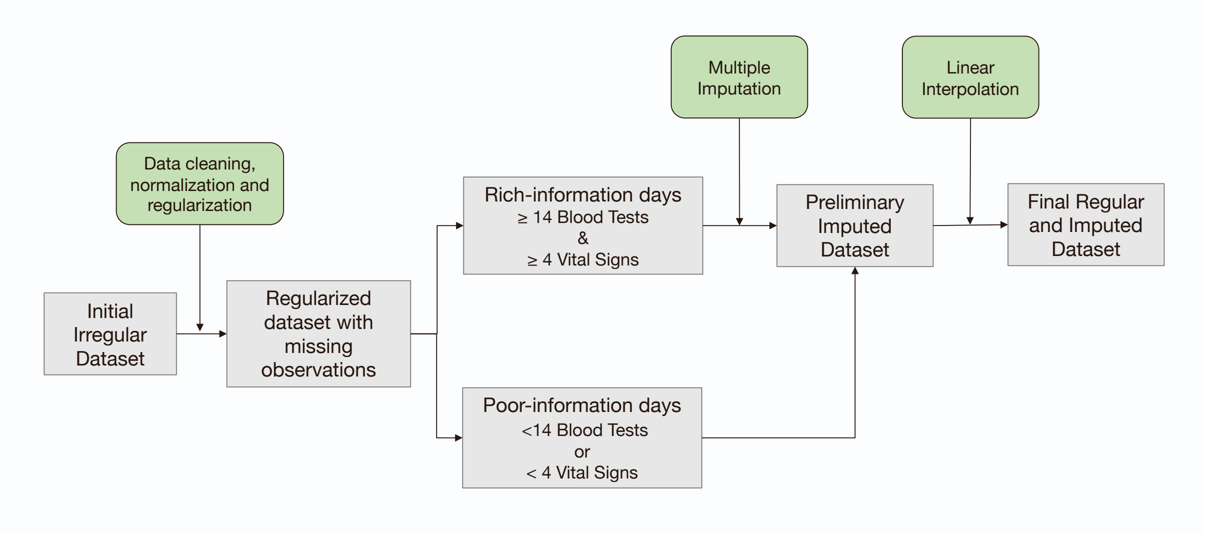

Figure S20: Missingness information in the final dataset. A: Overall fraction of missingness by laboratory test results and vital signs (i.e., proportion of total with missing value for each variable); B: Correlation matrix between missingness patters for all variables; C: Correlation matrix between normalised values for all variables; D: Final predictors matrix used for multiple imputation. Related to STAR Methods.

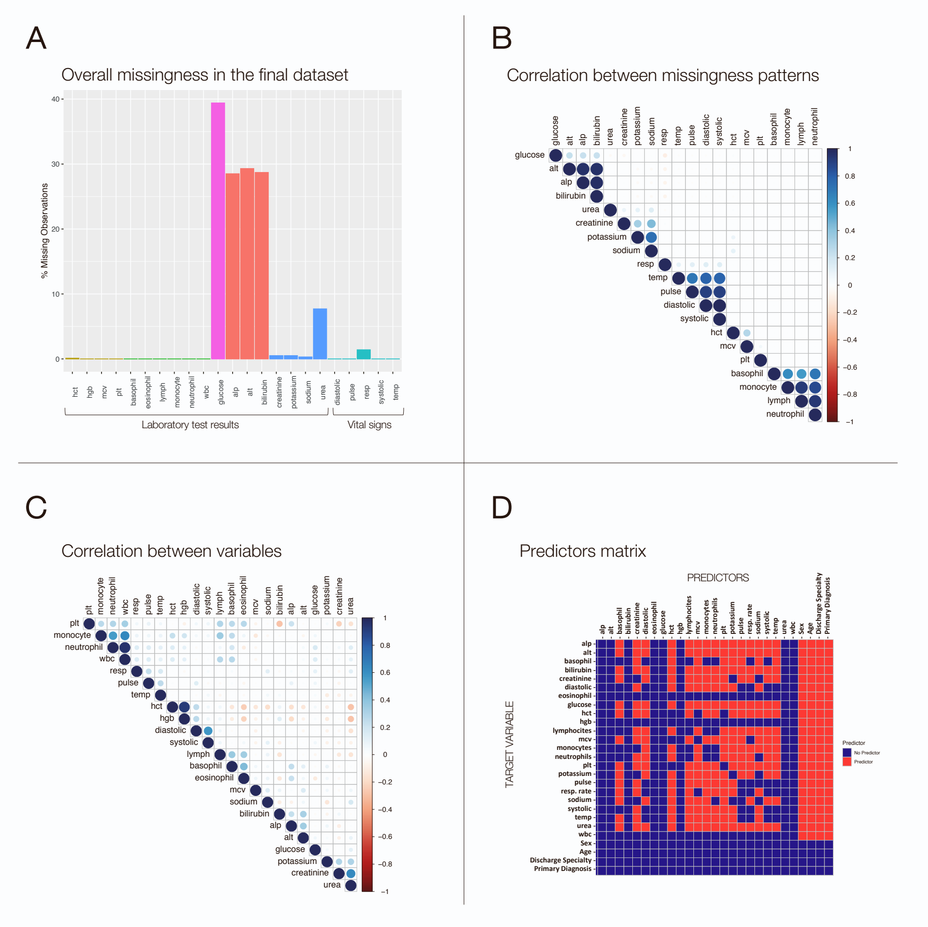

Figure S21: Evaluation of missingness imputation using Multiple Imputation and Linear Interpolation imputation methods. Related to STAR Methods.

A

Multiple Imputation:

Distribution of original (blue) and imputed (red) values for 10 imputed datasets

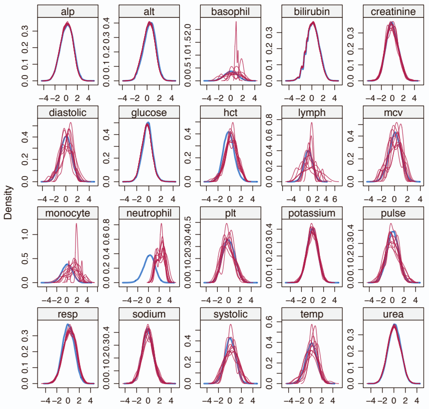

Number of missing values per variable				
23117	23715	**4**	23270	429
**22**	31918	53	**7**	**11**
**6**	**8**	**35**	394	27
1130	226	**22**	**36**	6241

B

Multiple Imputation:

Converge plots for multiple iterations

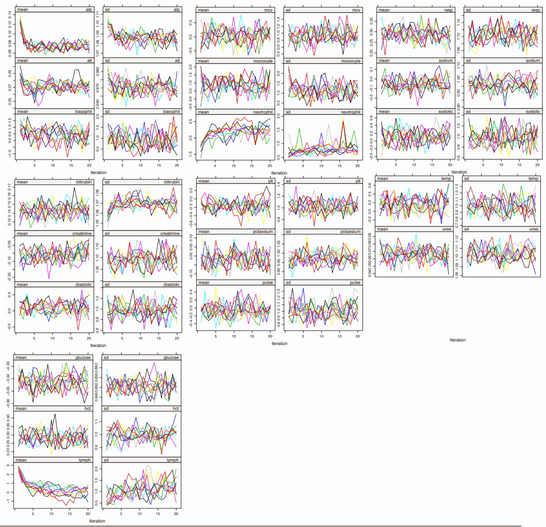

C

Multiple Imputation:

Distribution of randomly inserted (green) vs originally missing (blue) and non-missing (red) values

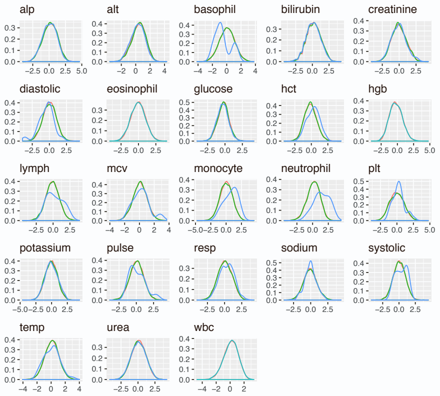

D

Linear Interpolation Imputation:

Distribution of randomly inserted (green) vs originally missing (blue), non-missing (red) and MI imputed (purple)values

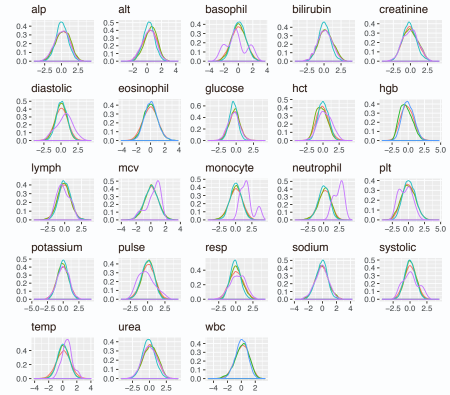

Table S1: Summary of patients' characteristics in the final cohort divided as 'training and validation' (80 % of the patients) and 'hold-out test' (20% of the patients) sets. The analyses and results in this publication are conducted only with the training and test set while the validation is kept apart for future confirmatory analyses. Related to Figure 1 and STAR Methods.

Characteristics		Training and validation	Test
Number of patients		8926	2232
Sex (%)	Women	47.2	48.2
Age-band (%)	65-69	20.9	19.1
	70-74	19.8	21.9
	75-89	19.2	19.2
	80-84	17.9	18.5
	85-89	13.3	12.7
	90-94	7	6.5
	95-99	1.8	1.9
	>=100	0.1	0.2
Discharge Specialty (%)	Geriatric Medicine	14.9	14.3
	Other Medicine	51.8	53.5
	Surgery	33.2	32.2
Clinical Frailty Scale Score at admission (%)	Up to Vulnerable	21.6	21.3
	Mildly Frail	7.9	8.1
	Moderately Frail	7.8	7.3
	Severe-Very Severely Frail/ Terminally Ill	4	4.6
	Not recorded	58.7	58.7
Length of Stay, days (%)	<=3	28.1	29.8
	4-5	20.7	19.4
	6-10	26	25.8
	>=11	25.1	25
Number of diagnoses recorded at discharge (%)	1-3	34.3	35
	4-5	20.8	21.1
	6-8	20.4	18.4
	>=9	15.1	15.3
	Not recorded	9.4	10.2
Inpatient Mortality (%)	Inpatient Death	8.4	7.7
	Discharged Alive	91.6	92.3
30-day post-discharge readmission (%)	Readmitted	14	15.1
30-day post-discharge mortality (%)	Recorded Death	3.2	3.1
Primary diagnosis at admission as top-level ICD-10 codes (%)	A	6.9	7.7
	B	0.3	0.7
	C	5.4	5.2
	D	1.9	1.9
	E	2.4	2.6
	F	0.7	0.9
	G	1.6	1.8
	H	0.2	0.1
	I	16.2	15.9
	J	14.5	13.7
	K	16.5	17.4
	L	1.8	1.7
	M	2.3	1.9
	N	8.9	8
	Q	0.1	0.1
	R	8.3	8.1
	S	9.8	9.6
	T	2.3	2.5
	Z	0.1	0.04

Table S2: Clinical interpretation of *Disease-Like* and *Admission-Like* HMM states, related to Table 1 (Predominant features highlighted in green)

States		Features			Hospital Outcome
		**Pattern of laboratory and physiological abnormalities**	**Association with Primary Diagnosis**	**Temporal relationship with Admission Episode (AE)**	**Association with LOS/ Inpatient death**
DISEASE-LIKE	Hepatic	High LFTs	**Digestive system (K) and neoplasms (C)**	Throughout AE	Long LOS
	Stable renal	High creatinine and urea; low haemaglobin and haematocrit	**Genitourinary system (N)**	Throughout AE	Long LOS
	Unstable renal	High creatinine and urea; low haemaglobin and haematocrit	**Genitourinary system (N) (and other diagnostic codes to a lesser extent)**	Throughout AE	Inpatient death
	Stable but static renal	Low haemoglobin and haematocrit; mildly high urea and creatinine; normal vital signs and WBC	Present in most diagnostic codes	Mid-late stages of the AE	Long LOS<br>Discharge alive
	Blood dyscrasia	All FBC parameters deranged in both directions with wide variance for WBC counts	**Neoplasms (C) and diseases of the blood (D)**	Throughout AE	-
	Bone marrow suppression	All FBC parameters low except MCV which is high	**Diseases of the blood (D) and neoplasms (C)**	Throughout AE	-
ADMISSION-LIKE	Acute presentation	High haemoglobin, haematocrit, total WBC, neutrophils and vital signs	Present in all diagnostic codes	**Strongly overrepresented on Day 1 of the AE, regardless of inpatient death or LOS**	-
	Treatment response (1)	Similar to acute presentation state but all parameters nearer group mean	Present in all diagnostic codes	**Day 2 to Day 8 (or discharge) of the AE**	Short LOS<br>Discharge alive
	Treatment response (2)	Low lymphocytes. All other parameters near group mean.	Present in most diagnostic codes except genitourinary (N) and symptoms/ signs/ clinical findings (R)	**Day 2 onwards but usually resolved by discharge if discharged alive**	Inpatient death
	Early discharge	All parameters near group mean. Where slight deviations occur, these tend to be in directions that would be closer to a 'healthy population' mean	Present in all diagnostic codes	**Last days of short AEs**	Short LOS<br>Discharge alive
	Pre-discharge	Low creatinine and urea; high platelets; other parameters near group mean	Present in all diagnostic codes	**Last days of long AEs, more often if discharged alive**	Long LOS<br>Discharge alive

LOS: length of stay; LFTs: liver function tests; FBC: full blood count; WBC: white blood cells; MCV: mean cell volume; Not all states could be classified as confidently after initial visual clinical interpretation (either no or >1 predominant feature)

Table S3: Clinical interpretation of *Physiological-Like* HMM states, related to Table 1 (Predominant features highlighted in green)

States		Features			Hospital Outcome
		Pattern of laboratory and physiological abnormalities	Association with Primary Diagnosis	Temporal relationship with Admission Episode (AE)	Association with LOS/ Inpatient death
PHYSIOLOGICAL-LIKE	Early inflammatory response	**Markedly high WBC and neutrophils; high urea, respiratory rate, and heart rate**	Present in all diagnostic codes	Throughout AE	Inpatient death
	Resolving inflammatory response	**High WBC, neutrophils and platelets and all other parameters either higher or lower compared to group mean, except for respiratory rate**	Present in infectious diseases (A), neoplasms (C), circulatory (I), respiratory (J), and digestive systems (K)	**Throughout AEs although more common after Day 1**	Long LOS<br>Discharge alive
	Autoimmune/ atopic	**Markedly higher basophils, eosinophils and lymphocytes**	Uncommon in all diagnostic codes	Throughout AE	Long LOS
	Acute thrombotic	**High haemoglobin, haematocrit and lymphocytes**	Present in all diagnostic codes and especially in Diseases of the circulatory system (I) and symptoms/ signs/ clinical findings (R)	Throughout AE	Short LOS<br>Discharge alive
	Prolonged illness	**Most parameters higher than group mean (especially respiratory rate) except haemoglobin, haematocrit and urea which are lower**	Present in most diagnostic codes except Genitourinary system (N) and symptoms/ signs/ clinical findings (R)	**More common after Day 4-5**	Long LOS
	Other illness presentation	All parameters at or near group mean	Present in most diagnostic codes with slight preference for Endocrine, nutritional and metabolic diseases (E)	More common in first 3-4 days of AE	Short LOS

LOS: length of stay; LFTs: liver function tests; FBC: full blood count; WBC: white blood cells; MCV: mean cell volume; Not all states could be classified as confidently after initial visual clinical interpretation (either no or >1 predominant feature)

Table S4: Variables retrieved from Electronic health Record (Epic) system. Related to STAR Methods

The NHS number and local patient identifiers are replaced by a pseudonym that is consistent for each patient. Information extracted is outlined in the table below, including how the information is converted during the anonymisation process to ensure the collective data for each patient is not identifiable.

**Information for extraction**	**Conversion of potentially sensitive information**
Hospital number (patient identifier)	Anonymised unique patient study code
Admission ID (identifier for each admission)	Anonymised unique admission study code
Sex	M/F
Date of birth	5-year age bands from 65 to >100 at admission
Physiological data (heart rate, blood pressure, respiratory rate, oxygen saturation, temp, MEWS-Score)	n/a<br>*Dates provided as consecutive days using date of admission as Day 1.*<br>*Times are maintained as recorded in EPIC*
Relevant blood biochemistry	Liver function, renal function, clotting factor and blood glucose, full blood count
Frailty scores	n/a
Medication history	n/a
Date of admission	Month of admission
Admission specialty	Broad category (Department of Medicine for the Elderly, Medicine or Surgery)
Number of ward moves	0,1,2,3,4,5,6,7,8,>8
Length of stay	1,2,3,4,5,6,7,8,9,10, ≥11
Discharge to usual place of residence/ new institutionalization	n/a<br>Yes/No
Diagnostic codes at discharge	High level ICD-10 codes (I-XXII)
Discharge specialty	Broad category (Department of Medicine for the Elderly, Medicine or Surgery)
Inpatient mortality (death during this inpatient episode)	Yes/No
Death within 30 days of discharge	Yes/No
Readmission within 30 days after discharge	Yes/No
Delayed transfer of care (define as more than 24 hours after last recorded clinically fit date)	Yes/No

Table S5. Hypotheses tested for prediction of clinical outcomes with discriminative models. Related to STAR Methods.

Model	Representation	Covariates	Input variables	Outcome			
				Inpatient Mortality	Clinical outcome at 30-days	Diagnosis at Admission	Diagnosis at Discharge
LR	Multivariate time series	DM	D1	x			
			D2	x			
			D3	x			
			D1D2	x			
			D2D3	x			
			D1D2D3	x			
		DM + PDA	D1	x			
			D2	x			
			D3	x			
			D1D2	x			
			D2D3	x			
			D1D2D3	x			
LR	HMM states	DM	D1	x			
			D2	x			
			D3	x			
			D1D2	x			
			D2D3	x			
			D1D2D3	x			
		DM + PDA	D1	x			
			D2	x			
			D3	x			
			D1D2	x			
			D2D3	x			
			D1D2D3	x			
RF	Multivariate time series	DM	D1	x	x	x	x
			D2	x	x	x	x
			D3	x	x	x	x
			D1D2	x	x	x	x
			D2D3	x	x	x	x
			D1D2D3	x	x	x	x
		DM + PDA	D1	x	x		
			D2	x	x		
			D3	x	x		
			D1D2	x	x		
			D2D3	x	x		
			D1D2D3	x	x		
RF	HMM states	DM	D1	x	x	x	x
			D2	x	x	x	x
			D3	x	x	x	x
			D1D2	x	x	x	x
			D2D3	x	x	x	x
			D1D2D3	x	x	x	x
		DM + PDA	D1	x	x		
			D2	x	x		
			D3	x	x		
			D1D2	x	x		
			D2D3	x	x		
			D1D2D3	x	x		

Table S6. ROC-AUC results for prediction of inpatient mortality as mean (SD) in the *training and validation* dataset and in the *hold-out test* dataset. Related to Table 2

Training and validation					
		RF		LR	
		MVTS	STATES	MVTS	STATES
NOPDA	D1	0.757 (0.001)	0.686 (0.001)	0.732 (0)	0.698 (0)
	D2	0.799 (0.001)	0.737 (0.001)	0.785 (0)	0.744 (0)
	D3	0.844 (0.001)	**0.778 (0)**	0.825 (0)	**0.782 (0)**
	D1D2	0.814 (0.001)	0.742 (0)	0.803 (0)	0.744 (0)
	D2D3	0.841 (0.001)	0.764 (0.001)	0.826 (0)	0.776 (0)
	D1D2D3	**0.848 (0.001)**	0.765 (0.001)	**0.832 (0)**	0.774 (0)
PDA	D1	0.77 (0.002)	0.728 (0.001)	0.746 (0)	0.729 (0)
	D2	0.81 (0.003)	0.764 (0)	0.793 (0)	0.76 (0)
	D3	**0.851 (0.002)**	**0.792 (0.001)**	0.829 (0)	**0.791 (0**)
	D1D2	0.816 (0.001)	0.762 (0.001)	0.805 (0)	0.759 (0)
	D2D3	0.844 (0.001)	0.78 (0)	0.828 (0)	0.786 (0)
	D1D2D3	0.847 (0.002)	0.778 (0.001)	**0.833 (0)**	0.786 (0)
Hold-out test					
INPUT VARIABLES		RF		LR	
		MVTS	STATES	MVTS	STATES
NOPDA	D1	0.781 (0.002)	0.632 (0.001)	0.755 (0)	**0.639 (0)**
	D2	0.791 (0.002)	0.623 (0.001)	0.785 (0)	0.624 (0)
	D3	**0.853 (0.001)**	0.575 (0.002)	0.834 (0)	0.559 (0)
	D1D2	0.815 (0.002)	**0.633 (0.002)**	0.814 (0)	0.556 (0)
	D2D3	0.849 (0.001)	0.583 (0.001)	0.839 (0)	0.575 (0)
	D1D2D3	**0.86 (0.001)**	0.586 (0.005)	**0.859 (0)**	0.529 (0)
PDA	D1	0.801 (0.003)	**0.683 (0.002)**	0.789 (0)	**0.691 (0)**
	D2	0.81 (0.001)	0.671 (0.002)	0.822 (0)	0.679 (0)
	D3	**0.863 (0.001)**	0.632 (0.005)	0.853 (0)	0.627 (0)
	D1D2	0.822 (0.002)	0.669 (0.004)	0.838 (0)	0.622 (0)
	D2D3	0.856 (0.002)	0.63 (0.002)	0.856 (0)	0.634 (0)
	D1D2D3	**0.862 (0.002)**	0.63 (0.004)	**0.869 (0)**	0.625 (0)

Table S7. Precision-Recall Curve AUC results for prediction of inpatient mortality as mean (SD) in the *training and validation* dataset and in the *hold-out test* dataset. Related to Table 2

Training and validation dataset					
INPUT VARIABLES		RF		LR	
		MVTS	STATES	MVTS	STATES
NO PDA	D1	0.242 (0.002)	0.166 (0.001)	0.217 (0)	0.178 (0)
	D2	0.328 (0.004)	0.212 (0.003)	0.279 (0)	0.212 (0)
	D3	**0.378 (0.003)**	**0.248 (0.003)**	0.371 (0)	**0.252 (0)**
	D1D2	0.32 (0.004)	0.204 (0.001)	0.315 (0)	0.208 (0)
	D2D3	0.353 (0.003)	0.231 (0.002)	0.367 (0)	0.244 (0)
	D1D2D3	0.368 (0.002)	0.22 (0.001)	**0.39 (0)**	0.23 (0)
PDA	D1	0.257 (0.003)	0.221 (0.002)	0.232 (0)	0.193 (0)
	D2	0.34 (0.005)	0.259 (0.001)	0.295 (0)	0.236 (0)
	D3	**0.392 (0.005)**	0.29 (0.003)	0.369 (0)	**0.27 (0)**
	D1D2	0.314 (0.004)	0.24 (0.005)	0.323 (0)	0.225 (0)
	D2D3	0.364 (0.003)	**0.268 (0.001)**	0.364 (0)	0.255 (0)
	D1D2D3	0.36 (0.005)	0.255 (0.002)	**0.395 (0)**	0.25 (0)
Hold-out test					
INPUT VARIABLES		RF		LR	
		MVTS	STATES	MVTS	STATES
NO PDA	D1	0.209 (0.002)	**0.125 (0.001)**	0.209 (0)	**0.167 (0)**
	D2	0.265 (0.005)	0.113 (0.001)	0.303 (0)	0.134 (0)
	D3	**0.364 (0.002)**	0.089 (0.001)	0.381 (0)	0.099 (0)
	D1D2	0.269 (0.004)	0.115 (0.001)	0.34 (0)	0.104 (0)
	D2D3	0.356 (0.003)	0.088 (0)	0.397 (0)	0.101 (0)
	D1D2D3	0.355 (0.005)	0.09 (0.001)	**0.422 (0)**	0.085 (0)
PDA	D1	0.225 (0.005)	**0.169 (0.004)**	0.236 (0)	**0.179 (0)**
	D2	0.276 (0.007)	0.133 (0.004)	0.329 (0)	0.145 (0)
	D3	**0.381 (0.004)**	0.107 (0.003)	0.411 (0)	0.117 (0)
	D1D2	0.265 (0.003)	0.13 (0.003)	0.354 (0)	0.116 (0)
	D2D3	0.361 (0.005)	0.104 (0.002)	0.422 (0)	0.124 (0)
	D1D2D3	0.341 (0.004)	0.104 (0.002)	**0.438 (0)**	0.118 (0)

Table S8. Precision, Recall and F1-scores results for prediction of inpatient mortality as mean (SD) in the *training and validation* dataset. Related to Table 2

PRECISION					
INPUT VARIABLES		RF		LR	
		MVTS	STATES	MV TS	STATES
NO PDA	D1	0.018 (0)	0.018 (0)	0.018 (0)	0.018 (0)
	D2	0.032 (0)	0.032 (0)	0.032 (0)	0.032 (0)
	D3	0.596 (0.014)	**0.188 (0.001)**	0.212 (0)	0.172 (0)
	D1D2	0.5 (0.04)	0.17 (0.001)	0.2 (0)	0.16 (0)
	D2D3	0.603 (0.031)	0.184 (0.002)	0.218 (0)	0.17 (0)
	D1D2D3	**0.617 (0.028)**	0.179 (0.002)	**0.223 (0)**	**0.176 (0)**
PDA	D1	0.675 (0.026)	0.173 (0.001)	0.176 (0)	0.167 (0)
	D2	**1 (0)**	0.18 (0.001)	0.196 (0)	0.166 (0)
	D3	0.718 (0.049)	**0.199 (0.002)**	**0.215 (0)**	**0.191 (0)**
	D1D2	0 (0)	0.188 (0.003)	0.208 (0)	0.174 (0)
	D2D3	0.523 (0.139)	0.19 (0.001)	0.214 (0)	0.183 (0)
	D1D2D3	0.571 (0.053)	0.191 (0.003)	0.223 (0)	0.181 (0)
RECALL					
INPUT VARIABLES		RF		LR	
		MVTS	STATES	MVTS	STATES
NO PDA	D1	0.018 (0)	0.556 (0)	0.673 (0)	0.606 (0)
	D2	0.032 (0)	0.647 (0)	0.704 (0)	0.686 (0)
	D3	**0.167 (0)**	**0.701 (0)**	**0.743 (0)**	**0.726 (0)**
	D1D2	0.026 (0)	0.623 (0)	0.73 (0)	0.721 (0)
	D2D3	0.054 (0)	0.678 (0)	0.735 (0)	**0.726 (0)**
	D1D2D3	0.092 (0)	0.677 (0)	**0.743 (0)**	0.712 (0)
PDA	D1	0.009 (0)	0.581 (0)	0.668 (0)	0.664 (0)
	D2	0.004 (0)	0.623 (0)	0.717 (0)	0.681 (0)
	D3	0.047 (0)	**0.693 (0)**	0.712 (0)	**0.752 (0)**
	D1D2	0 (0)	0.621 (0)	0.735 (0)	0.708 (0)
	D2D3	0.011 (0)	0.678 (0)	0.704 (0)	0.73 (0)
	D1D2D3	0.046 (0)	0.679 (0)	**0.739 (0)**	0.708 (0)
F1-SCORE					
INPUT VARIABLES		RF		LR	
		MVTS	STATES	MVTS	STATES
NO PDA	D1	0.035 (0.005)	0.246 (0.002)	0.271 (0)	0.222 (0)
	D2	0.06 (0.014)	0.26 (0.002)	0.288 (0)	0.25 (0)
	D3	**0.261 (0.01)**	**0.296 (0.002)**	0.329 (0)	0.278 (0)
	D1D2	0.05 (0.005)	0.267 (0.002)	0.314 (0)	0.262 (0)
	D2D3	0.099 (0.009)	0.29 (0.002)	0.336 (0)	0.275 (0)
	D1D2D3	0.161 (0.008)	0.283 (0.002)	**0.343 (0)**	**0.282 (0)**
PDA	D1	0.018 (0.003)	0.267 (0.002)	0.279 (0)	0.267 (0)
	D2	0.009 (0)	0.279 (0.002)	0.308 (0)	0.266 (0)
	D3	**0.089 (0.008)**	**0.309 (0.002)**	0.33 (0)	**0.305 (0)**
	D1D2	0 (0)	0.289 (0.004)	0.325 (0)	0.279 (0)
	D2D3	0.022 (0.01)	0.297 (0.002)	0.329 (0)	0.292 (0)
	D1D2D3	0.084 (0.011)	0.298 (0.004)	**0.343 (0)**	0.289 (0)

Table S9. Precision, Recall and F1-scores results for prediction of inpatient mortality as mean (SD) in the *hold-out test* dataset. Related to Table 2

PRECISION					
INPUT VARIABLES		RF		LR	
		MVTS	STATES	MV TS	STATES
NO PDA	D1	0.252 (0.049)	0.133 (0.001)	0.158 (0)	0.117 (0)
	D2	0.623 (0.07)	0.12 (0.003)	0.162 (0)	**0.119 (0)**
	D3	0.547 (0.019)	0.092 (0.003)	0.214 (0)	0.092 (0)
	D1D2	0.489 (0.092)	**0.135 (0.004)**	0.191 (0)	0.085 (0)
	D2D3	**0.802 (0.078)**	0.082 (0.002)	0.218 (0)	0.097 (0)
	D1D2D3	0.69 (0.042)	0.089 (0.004)	**0.227 (0)**	0.081 (0)
PDA	D1	0.1 (0.316)	0.137 (0.002)	0.177 (0)	0.131 (0)
	D2	0 (0)	0.136 (0.002)	0.191 (0)	**0.135 (0)**
	D3	0.751 (0.048)	0.121 (0.004)	0.212 (0)	0.114 (0)
	D1D2	0 (0)	**0.144 (0.004)**	0.202 (0)	0.099 (0)
	D2D3	**0.9 (0.316)**	0.12 (0.003)	0.22 (0)	0.11 (0)
	D1D2D3	0.708 (0.114)	0.125 (0.005)	**0.238 (0)**	0.11 (0)
RECALL					
INPUT VARIABLES		RF		LR	
		MVTS	STATES	MVTS	STATES
NO PDA	D1	0.006 (0)	**0.485 (0.004)**	0.667 (0)	0.526 (0)
	D2	0.034 (0.006)	0.377 (0.007)	0.684 (0)	0.485 (0)
	D3	**0.151 (0.005)**	0.364 (0.019)	0.749 (0)	0.503 (0)
	D1D2	0.016 (0.002)	0.452 (0.02)	0.713 (0)	**0.637 (0)**
	D2D3	0.051 (0.008)	0.298 (0.006)	0.743 (0)	0.503 (0)
	D1D2D3	0.079 (0.003)	0.329 (0.021)	**0.76 (0)**	0.561 (0)
PDA	D1	0.001 (0.002)	**0.526 (0.005)**	0.725 (0)	0.532 (0)
	D2	0 (0)	0.414 (0.013)	0.76 (0)	0.526 (0)
	D3	**0.051 (0.007)**	0.456 (0.022)	0.719 (0)	0.532 (0)
	D1D2	0 (0)	0.462 (0.018)	0.743 (0)	**0.632 (0)**
	D2D3	0.007 (0.004)	0.395 (0.01)	0.725 (0)	0.515 (0)
	D1D2D3	0.032 (0.006)	0.401 (0.03)	**0.778 (0)**	0.596 (0)
F1-SCORE					
INPUT VARIABLES		RF		LR	
		MVTS	STATES	MVTS	STATES
NO PDA	D1	0.011 (0)	**0.208 (0.001)**	0.256 (0)	**0.192 (0)**
	D2	0.064 (0.011)	0.182 (0.004)	0.262 (0)	0.191 (0)
	D3	**0.237 (0.008)**	0.146 (0.005)	0.332 (0)	0.156 (0)
	D1D2	0.032 (0.005)	**0.208 (0.004)**	0.301 (0)	0.15 (0)
	D2D3	0.097 (0.014)	0.128 (0.003)	**0.337 (0)**	0.163 (0)
	D1D2D3	0.142 (0.006)	0.14 (0.006)	0.35 (0)	0.141 (0)
PDA	D1	0.001 (0.004)	0.217 (0.003)	0.285 (0)	0.21 (0)
	D2	0 (0)	0.204 (0.004)	0.305 (0)	**0.214 (0)**
	D3	**0.095 (0.012)**	0.191 (0.006)	0.327 (0)	0.188 (0)
	D1D2	0 (0)	**0.219 (0.004)**	0.318 (0)	0.171 (0)
	D2D3	0.014 (0.007)	0.184 (0.005)	0.338 (0)	0.181 (0)
	D1D2D3	0.061 (0.012)	0.191 (0.007)	**0.365 (0)**	0.186 (0)

Table S10. Weighted ROC-AUC for prediction of 30-day clinical outcome in the *training and validation* dataset and in the *hold-out test* dataset. Related to Table 2

Training and validation			
INPUT VARIABLES		RF	
		MVTS	STATES
NO PDA	D1	0.634 (0.001)	0.588 (0)
	D2	0.638 (0)	0.625 (0.001)
	D3	0.66 (0)	0.645 (0.001)
	D1D2	0.653 (0.001)	0.628 (0.001)
	D2D3	0.664 (0)	**0.647 (0)**
	D1D2D3	**0.674 (0.001)**	**0.647 (0)**
PDA	D1	0.649 (0)	0.636 (0.001)
	D2	0.654 (0.001)	0.654 (0)
	D3	0.679 (0.001)	**0.671 (0)**
	D1D2	0.661 (0.001)	0.651 (0)
	D2D3	0.673 (0.001)	0.667 (0)
	D1D2D3	**0.681 (0.001)**	0.664 (0)
Hold-out test			
INPUT VARIABLES		RF	
		MVTS	STATES
NO PDA	D1	0.629 (0.001)	0.553 (0.001)
	D2	0.64 (0.001)	0.552 (0.001)
	D3	0.664 (0)	0.54 (0.001)
	D1D2	0.657 (0.001)	**0.558 (0.001)**
	D2D3	0.668 (0.001)	0.546 (0.001)
	D1D2D3	**0.676 (0)**	0.555 (0.002)
PDA	D1	0.641 (0.001)	**0.592 (0.002)**
	D2	0.656 (0)	0.585 (0.001)
	D3	0.676 (0.001)	0.575 (0.001)
	D1D2	0.663 (0.001)	0.585 (0.001)
	D2D3	0.676 (0.001)	0.577 (0.001)
	D1D2D3	**0.681 (0.001)**	0.582 (0.001)

Table S11. Weighted precision, recall and F1-score for prediction of 30-day clinical outcome in the *training and validation* dataset and in the *hold-out test* dataset. Related to Table 2

Training and validation							
INPUT VARIABLES		RF					
		Precision		Recall		F1-score	
		MVTS	MV TS	MVTS	STATES	MVTS	STATES
NO PDA	D1	0.64 (0.002)	0.625 (0)	0.662 (0.002)	0.578 (0.002)	0.648 (0.002)	0.576 (0)
	D2	0.647 (0.002)	0.641 (0.001)	0.651 (0.002)	**0.592 (0.005)**	0.646 (0.001)	0.591 (0.003)
	D3	0.659 (0.001)	0.651 (0.002)	0.68 (0.002)	0.591 (0.006)	**0.669 (0.002)**	**0.593 (0.005)**
	D1D2	0.651 (0.002)	0.64 (0.002)	0.672 (0.002)	0.582 (0.007)	0.656 (0.002)	0.588 (0.004)
	D2D3	**0.66 (0.001)**	**0.655 (0.001)**	0.685 (0.002)	0.574 (0.002)	0.668 (0.001)	0.585 (0.002)
	D1D2D3	**0.66 (0.004)**	0.652 (0.001)	**0.692 (0.002)**	0.58 (0.003)	**0.669 (0.002)**	0.588 (0.001)
PDA	D1	0.653 (0.003)	0.645 (0.001)	0.65 (0.002)	0.618 (0.005)	0.646 (0.002)	0.611 (0.003)
	D2	0.652 (0.003)	0.651 (0.001)	0.641 (0.002)	**0.632 (0.005)**	0.645 (0.002)	**0.62 (0.003)**
	D3	0.661 (0.001)	0.655 (0.001)	0.67 (0.002)	0.624 (0.003)	0.665 (0.001)	0.617 (0.002)
	D1D2	0.655 (0.004)	0.649 (0.001)	0.667 (0.003)	0.621 (0.001)	0.655 (0.002)	0.608 (0.001)
	D2D3	0.66 (0.002)	**0.658 (0.001)**	0.678 (0.003)	0.604 (0.002)	0.665 (0.002)	0.607 (0.001)
	D1D2D3	**0.662 (0.002)**	0.657 (0.002)	**0.688 (0.002)**	0.606 (0.002)	**0.668 (0.001)**	0.597 (0.001)
Hold-out test							
INPUT VARIABLES		RF					
		Precision		Recall		F1-score	
		MVTS	MV TS	MVTS	STATES	MVTS	STATES
NO PDA	D1	0.634 (0.002)	0.612 (0.001)	0.659 (0.002)	**0.615 (0.006)**	0.644 (0.001)	**0.61 (0.003)**
	D2	0.633 (0.002)	0.61 (0.001)	0.65 (0.002)	0.542 (0.004)	0.639 (0.002)	0.556 (0.003)
	D3	**0.651 (0.001)**	0.601 (0.002)	0.684 (0.002)	0.493 (0.011)	**0.665 (0.001)**	0.517 (0.007)
	D1D2	0.636 (0.003)	**0.613 (0.002)**	0.672 (0.002)	0.586 (0.012)	0.648 (0.002)	0.594 (0.007)
	D2D3	0.654 (0.002)	0.612 (0.002)	0.686 (0.001)	0.479 (0.01)	0.664 (0.001)	0.511 (0.007)
	D1D2D3	0.649 (0.002)	0.611 (0.002)	**0.695 (0.002)**	0.55 (0.01)	**0.665 (0.001)**	0.569 (0.006)
PDA	D1	0.637 (0.001)	0.622 (0.002)	0.649 (0.002)	**0.639 (0.01)**	0.642 (0.002)	**0.623 (0.005)**
	D2	0.64 (0.001)	0.619 (0.001)	0.647 (0.002)	0.617 (0.006)	0.64 (0.001)	0.602 (0.004)
	D3	**0.657 (0.002)**	0.615 (0.001)	0.681 (0.002)	0.557 (0.004)	**0.666 (0.002)**	0.565 (0.002)
	D1D2	0.636 (0.001)	0.617 (0.001)	0.666 (0.002)	0.603 (0.005)	0.646 (0.001)	0.597 (0.003)
	D2D3	0.656 (0.002)	0.621 (0.001)	0.683 (0.002)	0.543 (0.004)	0.665 (0.002)	0.56 (0.003)
	D1D2D3	0.649 (0.002)	**0.624 (0.001)**	**0.691 (0.002)**	0.553 (0.008)	0.663 (0.002)	0.568 (0.005)

Table S12. Precision by class for prediction of 30-day clinical outcome in the *training and validation* dataset and in the *hold-out test dataset*. Related to Table 2

Precision in training and validation dataset											
INPUT VARIABLES		RF									
		MVTS					STATES				
		ID	DA	PDR	PDRM	PDM	ID	DA	PDR	PRDM	PDM
NO PDA	D1	0.318 (0.016)	0.786 (0.001)	0.161 (0.009)	0 (0)	**0.076 (0.019)**	0.134 (0.001)	0.793 (0)	0.133 (0.002)	0.008 (0)	0.04 (0.002)
	D2	0.326 (0.007)	0.795 (0.001)	0.159 (0.008)	0 (0)	0.067 (0.014)	0.174 (0.001)	0.806 (0.001)	0.155 (0.003)	0.011 (0)	0.047 (0.002)
	D3	0.371 (0.009)	0.803 (0.001)	0.184 (0.006)	0 (0)	0.049 (0.018)	**0.191 (0.001)**	0.815 (0.003)	**0.168 (0.005)**	0.014 (0.002)	**0.048 (0.002)**
	D1D2	0.384 (0.012)	0.795 (0.002)	0.163 (0.008)	0 (0)	0 (0)	0.182 (0.004)	0.803 (0.002)	0.154 (0.002)	0.014 (0.001)	0.037 (0.004)
	D2D3	0.381 (0.006)	**0.804 (0.001)**	0.18 (0.013)	0 (0)	0 (0)	0.19 (0.002)	**0.821 (0.001)**	**0.168 (0.002)**	0.011 (0.001)	0.039 (0.002)
	D1D2D3	**0.4 (0.008)**	0.799 (0.001)	**0.188 (0.015)**	0 (0)	0.039 (0.087)	0.19 (0.002)	0.816 (0.002)	0.166 (0.003)	0.008 (0.004)	0.044 (0.004)
PDA	D1	0.324 (0.007)	0.794 (0.001)	0.161 (0.009)	0 (0)	**0.368 (0.095)**	0.174 (0.003)	0.809 (0.001)	0.157 (0.002)	0.021 (0.001)	0.062 (0.003)
	D2	0.329 (0.004)	0.802 (0.001)	0.158 (0.008)	0 (0)	0.062 (0.043)	0.196 (0.003)	0.813 (0.001)	**0.169 (0.004)**	0.011 (0.011)	**0.064 (0.004)**
	D3	0.363 (0.009)	**0.807 (0.001)**	0.181 (0.005)	0 (0)	0 (0)	**0.209 (0.001)**	0.817 (0.001)	0.171 (0.003)	0.005 (0.006)	0.055 (0.003)
	D1D2	0.379 (0.012)	0.798 (0.001)	0.16 (0.009)	0 (0)	0.12 (0.14)	0.193 (0.001)	0.815 (0.001)	0.156 (0.002)	0.009 (0.002)	0.043 (0.001)
	D2D3	0.371 (0.011)	0.805 (0.001)	0.184 (0.008)	0 (0)	0 (0)	0.203 (0.001)	0.821 (0.001)	0.176 (0.003)	0.012 (0.001)	0.058 (0.004)
	D1D2D3	**0.408 (0.004)**	0.801 (0.001)	**0.196 (0.013)**	0 (0)	0 (0)	0.204 (0.001)	**0.823 (0.002)**	0.158 (0.003)	0.019 (0.002)	0.051 (0.002)

Precision in hold-out test dataset											
INPUT VARIABLES		RF									
		MVTS					STATES				
		ID	DA	PDR	PDRM	PDM	ID	DA	PDR	PRDM	PDM
NO PDA	D1	0.224 (0.009)	0.785 (0.001)	0.18 (0.006)	0 (0)	**0.123 (0.053)**	**0.143 (0.003)**	0.771 (0.001)	0.157 (0.001)	0 (0)	0.034 (0.001)
	D2	0.257 (0.006)	0.789 (0.001)	0.151 (0.009)	0 (0)	0 (0)	0.117 (0.003)	0.773 (0.002)	0.146 (0.002)	0.014 (0.001)	0.035 (0.001)
	D3	0.326 (0.005)	0.796 (0.001)	**0.2 (0.009)**	0 (0)	0.064 (0.009)	0.092 (0.003)	0.764 (0.003)	0.142 (0.004)	0.012 (0.001)	**0.036 (0.003)**
	D1D2	0.302 (0.011)	0.786 (0.001)	0.148 (0.015)	0 (0)	0.101 (0.111)	0.135 (0.004)	0.774 (0.001)	0.153 (0.008)	0.013 (0.008)	0.031 (0.002)
	D2D3	0.359 (0.004)	**0.797 (0.001)**	0.197 (0.009)	0 (0)	0.033 (0.07)	0.088 (0.003)	**0.779 (0.002)**	0.147 (0.001)	0.014 (0.001)	0.04 (0.003)
	D1D2D3	**0.373 (0.01)**	0.794 (0.001)	0.177 (0.01)	0 (0)	0 (0)	0.089 (0.004)	0.775 (0.002)	**0.161 (0.005)**	**0.019 (0.006)**	**0.036 (0.004)**
PDA	D1	0.234 (0.009)	0.791 (0.001)	0.18 (0.01)	0 (0)	0 (0)	**0.156 (0.005)**	0.779 (0.002)	**0.174 (0.008)**	0.015 (0.013)	0.064 (0.004)
	D2	0.268 (0.005)	0.795 (0.001)	0.165 (0.01)	0 (0)	0 (0)	0.135 (0.003)	0.778 (0.001)	0.167 (0.006)	**0.027 (0.017)**	**0.067 (0.003)**
	D3	0.335 (0.006)	**0.8 (0.001)**	**0.198 (0.005)**	0 (0)	**0.145 (0.062)**	0.123 (0.003)	0.776 (0.002)	0.16 (0.002)	0.017 (0.006)	0.063 (0.002)
	D1D2	0.307 (0.01)	0.787 (0.001)	0.155 (0.012)	0 (0)	0 (0)	0.147 (0.003)	0.775 (0.002)	0.171 (0.003)	0.013 (0.002)	0.042 (0.002)
	D2D3	0.372 (0.007)	0.799 (0.001)	**0.199 (0.011)**	0 (0)	0.02 (0.063)	0.109 (0.003)	0.786 (0.002)	0.159 (0.002)	0.009 (0.009)	0.059 (0.003)
	D1D2D3	**0.375 (0.011)**	0.795 (0.001)	0.169 (0.009)	0 (0)	0 (0)	0.105 (0.006)	**0.789 (0.001)**	0.165 (0.001)	0.015 (0)	0.041 (0.002)

Table S13. Recall by class for prediction of 30-day clinical outcome in the *training and validation* dataset and in the *hold-out test dataset*. Related to Table 2

Recall in training and validation dataset											
INPUT VARIABLES		RF									
		MVTS					STATES				
		ID	DA	PDR	PDRM	PDM	ID	DA	PDR	PRDM	PDM
NO PDA	D1	0.167 (0.009)	0.841 (0.002)	0.121 (0.009)	0 (0)	0.017 (0.005)	0.484 (0.003)	0.636 (0.001)	0.399 (0.016)	0.095 (0)	0.255 (0.013)
	D2	0.266 (0.007)	**0.822 (0.001)**	0.083 (0.006)	0 (0)	0.017 (0.005)	0.635 (0.011)	0.644 (0.006)	0.349 (0.015)	0.095 (0)	0.302 (0.013)
	D3	**0.329 (0.012)**	0.843 (0.002)	0.146 (0.005)	0 (0)	0.02 (0.007)	0.655 (0.008)	0.634 (0.009)	0.383 (0.013)	0.152 (0.02)	0.343 (0.013)
	D1D2	0.21 (0.006)	0.86 (0.002)	0.065 (0.003)	0 (0)	0 (0)	0.617 (0.014)	0.64 (0.009)	0.329 (0.015)	0.095 (0)	0.212 (0.034)
	D2D3	0.293 (0.005)	0.866 (0.002)	0.073 (0.004)	0 (0)	0 (0)	0.663 (0.005)	0.615 (0.004)	0.362 (0.007)	0.095 (0)	0.303 (0.016)
	D1D2D3	0.262 (0.01)	**0.882 (0.003)**	0.064 (0.005)	0 (0)	0.003 (0.006)	**0.669 (0.01)**	0.625 (0.003)	0.349 (0.008)	0.048 (0.022)	0.315 (0.035)
PDA	D1	0.188 (0.004)	0.823 (0.002)	0.12 (0.008)	0 (0)	0.025 (0.008)	0.577 (0.023)	0.686 (0.006)	0.346 (0.01)	0.095 (0)	0.306 (0.018)
	D2	0.299 (0.004)	0.806 (0.002)	0.082 (0.005)	0 (0)	0.011 (0.007)	0.669 (0.007)	0.692 (0.006)	0.36 (0.01)	0.043 (0.042)	0.306 (0.026)
	D3	**0.361 (0.007)**	0.826 (0.003)	0.147 (0.005)	0 (0)	0 (0)	0.68 (0.005)	0.679 (0.003)	0.371 (0.009)	0.019 (0.025)	0.291 (0.011)
	D1D2	0.223 (0.006)	0.852 (0.003)	0.065 (0.004)	0 (0)	0.009 (0.011)	0.675 (0.004)	0.667 (0.001)	0.424 (0.006)	0.11 (0.023)	0.26 (0.009)
	D2D3	0.297 (0.006)	0.856 (0.003)	0.079 (0.003)	0 (0)	0 (0)	0.673 (0.004)	0.654 (0.002)	0.369 (0.007)	0.048 (0)	0.289 (0.02)
	D1D2D3	0.272 (0.007)	**0.874 (0.001)**	0.067 (0.004)	0 (0)	0 (0)	**0.705 (0.006)**	0.632 (0.002)	0.461 (0.01)	0.219 (0.025)	0.365 (0.016)

Recall in hold-out test dataset											
INPUT VARIABLES		RF									
		MVTS					STATES				
		ID	DA	PDR	PDRM	PDM	ID	DA	PDR	PRDM	PDM
NO PDA	D1	0.137 (0.008)	0.838 (0.002)	**0.134 (0.005)**	0 (0)	**0.033 (0.016)**	0.282 (0.016)	**0.753 (0.007)**	0.175 (0.002)	0 (0)	0.169 (0.019)
	D2	0.251 (0.006)	0.828 (0.003)	0.069 (0.004)	0 (0)	0 (0)	0.353 (0.02)	0.611 (0.006)	0.356 (0.011)	**0.105 (0)**	0.245 (0.014)
	D3	**0.365 (0.006)**	0.85 (0.003)	0.128 (0.005)	0 (0)	0.02 (0)	**0.388 (0.019)**	0.542 (0.014)	0.352 (0.01)	**0.105 (0)**	0.241 (0.023)
	D1D2	0.212 (0.008)	0.864 (0.002)	0.053 (0.006)	0 (0)	0.018 (0.019)	0.311 (0.03)	0.707 (0.015)	0.191 (0.018)	0.047 (0.03)	0.214 (0.019)
	D2D3	0.323 (0.006)	0.868 (0.002)	0.073 (0.003)	0 (0)	0.004 (0.008)	0.325 (0.009)	0.522 (0.014)	**0.388 (0.007)**	**0.105 (0)**	**0.275 (0.013)**
	D1D2D3	0.285 (0.011)	**0.888 (0.003)**	0.054 (0.002)	0 (0)	0 (0)	0.295 (0.014)	0.648 (0.012)	0.247 (0.02)	0.074 (0.027)	0.245 (0.019)
PDA	D1	0.158 (0.007)	0.825 (0.003)	0.132 (0.009)	0 (0)	0 (0)	**0.418 (0.015)**	**0.76 (0.012)**	0.2 (0.013)	0.032 (0.027)	**0.384 (0.025)**
	D2	0.295 (0.009)	0.82 (0.003)	0.069 (0.004)	0 (0)	0 (0)	0.404 (0.015)	0.695 (0.009)	**0.405 (0.013)**	0.068 (0.043)	0.314 (0.013)
	D3	**0.392 (0.01)**	0.842 (0.003)	**0.136 (0.005)**	0 (0)	**0.018 (0.006)**	0.451 (0.008)	0.618 (0.005)	0.364 (0.004)	0.047 (0.017)	0.312 (0.011)
	D1D2	0.222 (0.01)	0.855 (0.003)	0.058 (0.005)	0 (0)	0 (0)	0.411 (0.015)	0.688 (0.008)	0.337 (0.007)	**0.158 (0.025)**	0.294 (0.013)
	D2D3	0.353 (0.006)	0.861 (0.003)	0.076 (0.004)	0 (0)	0.002 (0.006)	0.344 (0.011)	0.606 (0.006)	0.392 (0.007)	0.026 (0.028)	0.263 (0.017)
	D1D2D3	0.289 (0.011)	**0.882 (0.002)**	0.053 (0.002)	0 (0)	0 (0)	0.326 (0.023)	0.623 (0.01)	0.376 (0.01)	**0.158 (0)**	0.29 (0.012)

Table S14. F1-score by class for prediction of 30-day clinical outcome in the *training and validation* dataset and in the *hold-out test dataset*. Related to Table 2

F1-Score in training and validation dataset											
INPUT VARIABLES		RF									
		MVTS					STATES				
		ID	DA	PDR	PDRM	PDM	ID	DA	PDR	PRDM	PDM
NO PDA	D1	0.219 (0.011)	0.813 (0.001)	0.138 (0.009)	0 (0)	**0.028 (0.008)**	0.21 (0.001)	0.706 (0)	0.199 (0.004)	0.014 (0.001)	0.07 (0.003)
	D2	0.293 (0.007)	0.808 (0.001)	0.109 (0.007)	0 (0)	0.027 (0.007)	0.274 (0.001)	0.716 (0.003)	0.215 (0.004)	0.019 (0.001)	0.082 (0.003)
	D3	**0.349 (0.01)**	0.822 (0.001)	**0.162 (0.005)**	0 (0)	**0.028 (0.01)**	0.296 (0.002)	**0.713 (0.006)**	0.233 (0.005)	**0.026 (0.003)**	**0.085 (0.003)**
	D1D2	0.271 (0.007)	0.826 (0.002)	0.093 (0.004)	0 (0)	0 (0)	0.281 (0.004)	0.712 (0.006)	0.21 (0.004)	0.024 (0.001)	0.063 (0.008)
	D2D3	0.331 (0.004)	0.834 (0.001)	0.104 (0.006)	0 (0)	0 (0)	**0.296 (0.002)**	0.703 (0.002)	**0.23 (0.003)**	0.019 (0.001)	0.069 (0.004)
	D1D2D3	0.317 (0.009)	**0.838 (0.002)**	0.095 (0.007)	0 (0)	0.006 (0.012)	**0.296 (0.003)**	0.707 (0.001)	0.225 (0.003)	0.014 (0.007)	0.078 (0.007)
PDA	D1	0.238 (0.004)	0.808 (0.001)	0.137 (0.009)	0 (0)	**0.046 (0.015)**	0.267 (0.004)	0.742 (0.004)	0.216 (0.003)	**0.034 (0.002)**	0.104 (0.006)
	D2	0.313 (0.003)	0.804 (0.001)	0.108 (0.006)	0 (0)	0.018 (0.013)	0.304 (0.004)	**0.748 (0.004)**	0.23 (0.005)	0.017 (0.017)	**0.106 (0.007)**
	D3	**0.362 (0.007)**	0.817 (0.002)	**0.162 (0.004)**	0 (0)	0 (0)	**0.32 (0.002)**	0.742 (0.002)	0.234 (0.005)	0.007 (0.01)	0.093 (0.004)
	D1D2	0.28 (0.008)	0.824 (0.001)	0.093 (0.005)	0 (0)	0.017 (0.02)	0.3 (0.002)	0.734 (0.001)	0.228 (0.003)	0.017 (0.004)	0.073 (0.002)
	D2D3	0.33 (0.008)	0.829 (0.002)	0.111 (0.004)	0 (0)	0 (0)	0.312 (0.002)	0.728 (0.002)	**0.239 (0.004)**	0.019 (0.001)	0.096 (0.007)
	D1D2D3	0.327 (0.006)	**0.836 (0.001)**	0.1 (0.006)	0 (0)	0 (0)	0.316 (0.002)	0.715 (0.001)	0.235 (0.004)	**0.034 (0.004)**	0.09 (0.003)
F1-Score in hold-out test dataset											
INPUT VARIABLES		RF									
		MVTS					STATES				
		ID	DA	PDR	PDRM	PDM	ID	DA	PDR	PRDM	PDM
NO PDA	D1	0.17 (0.009)	0.811 (0.001)	0.153 (0.005)	0 (0)	**0.052 (0.025)**	0.19 (0.003)	**0.762 (0.004)**	0.166 (0.001)	0 (0)	0.056 (0.002)
	D2	0.254 (0.005)	0.808 (0.002)	0.094 (0.006)	0 (0)	0 (0)	0.175 (0.005)	0.683 (0.003)	0.207 (0.003)	0.024 (0.001)	0.062 (0.002)
	D3	**0.344 (0.004)**	0.822 (0.001)	**0.156 (0.006)**	0 (0)	0.03 (0.001)	0.149 (0.005)	0.634 (0.009)	0.203 (0.005)	0.022 (0.001)	**0.063 (0.006)**
	D1D2	0.249 (0.009)	0.823 (0.001)	0.078 (0.008)	0 (0)	0.03 (0.033)	**0.188 (0.007)**	0.739 (0.008)	0.17 (0.01)	0.021 (0.012)	0.055 (0.004)
	D2D3	**0.34 (0.005)**	0.831 (0.001)	0.107 (0.004)	0 (0)	0.007 (0.015)	0.139 (0.004)	0.625 (0.01)	**0.213 (0.002)**	**0.025 (0.001)**	0.07 (0.005)
	D1D2D3	0.323 (0.011)	**0.838 (0.002)**	0.083 (0.004)	0 (0)	0 (0)	0.136 (0.005)	0.706 (0.007)	0.195 (0.01)	0.03 (0.01)	**0.063 (0.007)**
PDA	D1	0.189 (0.008)	0.808 (0.001)	0.152 (0.009)	0 (0)	0 (0)	**0.227 (0.005)**	**0.769 (0.006)**	0.186 (0.01)	0.02 (0.017)	0.11 (0.007)
	D2	0.281 (0.007)	0.807 (0.002)	0.098 (0.006)	0 (0)	0 (0)	0.202 (0.005)	0.734 (0.005)	0.236 (0.008)	**0.038 (0.024)**	**0.111 (0.004)**
	D3	**0.362 (0.008)**	0.821 (0.002)	**0.161 (0.005)**	0 (0)	**0.031 (0.011)**	0.193 (0.004)	0.688 (0.003)	0.223 (0.002)	0.025 (0.009)	0.104 (0.004)
	D1D2	0.257 (0.01)	0.82 (0.001)	0.085 (0.007)	0 (0)	0 (0)	0.216 (0.004)	0.729 (0.004)	0.227 (0.004)	0.024 (0.003)	0.074 (0.003)
	D2D3	**0.362 (0.006)**	0.829 (0.002)	0.11 (0.005)	0 (0)	0.004 (0.011)	0.166 (0.005)	0.685 (0.004)	0.226 (0.003)	0.013 (0.014)	0.096 (0.006)
	D1D2D3	0.326 (0.011)	**0.836 (0.001)**	0.081 (0.003)	0 (0)	0 (0)	0.158 (0.009)	0.696 (0.006)	**0.229 (0.002)**	0.027 (0.001)	0.072 (0.003)

Table S15. Weighted ROC-AUC for prediction of primary diagnosis at admission (PDA) in the *training and validation* dataset. Related to Table 2

Training and validation			
INPUT VARIABLES		MVTS	STATES
NO PDA	D1	0.74 (0)	0.62 (0)
	D2	0.71 (0)	0.63 (0.001)
	D3	0.69 (0)	0.63 (0.001)
	D1D2	0.75 (0)	0.64 (0)
	D2D3	0.73 (0)	0.64 (0)
	D1D2D3	**0.76 (0)**	**0.65 (0)**
Hold-out test			
INPUT VARIABLES		MVTS	STATES
NO PDA	D1	0.732 (0)	0.54 (0.001)
	D2	0.696 (0)	0.532 (0.001)
	D3	0.686 (0)	0.537 (0.001)
	D1D2	0.738 (0)	0.537 (0.001)
	D2D3	0.715 (0)	0.539 (0.001)
	D1D2D3	**0.748 (0)**	**0.541 (0.001)**

Table S16. Weighted precision, recall and F1-score for prediction of primary diagnosis at admission (PDA) in the *training and validation* dataset. Related to Table 2

Training and validation							
INPUT VARIABLES		RF		RF		RF	
		Precision		Recall		F1-score	
		MVTS	STATES	MVTS	STATES	MVTS	STATES
NO PDA	D1	0.29 (0.002)	0.16 (0.001)	**0.39 (0.002)**	0.49 (0.005)	0.32 (0.002)	0.24 (0.002)
	D2	0.28 (0.002)	0.17 (0.001)	0.36 (0.002)	0.46 (0.002)	0.31 (0.002)	0.24 (0.001)
	D3	0.27 (0.002)	0.17 (0.001)	0.28 (0.002)	0.46 (0.007)	0.27 (0.002)	0.24 (0.002)
	D1D2	0.32 (0.001)	0.18 (0.002)	0.37 (0.002)	0.49 (0.004)	**0.34 (0.001)**	0.25 (0.002)
	D2D3	0.33 (0.002)	0.18 (0.001)	0.31 (0.002)	0.48 (0.003)	0.31 (0.002)	0.25 (0.001)
	D1D2D3	**0.35 (0.003)**	**0.19 (0.001)**	0.35 (0.002)	**0.5 (0.002)**	**0.34 (0.002)**	**0.26 (0.001)**
Hold-out test							
INPUT VARIABLES		RF		RF		RF	
		Precision		Recall		F1-score	
		MVTS	STATES	MVTS	STATES	MVTS	STATES
NO PDA	D1	0.29 (0.001)	**0.15 (0.003)**	**0.39 (0.001)**	0.27 (0.007)	0.32 (0.001)	0.17 (0.004)
	D2	0.27 (0.002)	0.12 (0.002)	0.34 (0.002)	0.3 (0.005)	0.3 (0.002)	0.16 (0.003)
	D3	0.27 (0.003)	0.13 (0.002)	0.28 (0.003)	0.32 (0.007)	0.27 (0.002)	0.18 (0.003)
	D1D2	0.32 (0.001)	0.14 (0.002)	0.37 (0.001)	0.26 (0.005)	**0.34 (0.001)**	0.16 (0.002)
	D2D3	0.32 (0.002)	0.13 (0.002)	0.3 (0.001)	**0.32 (0.004)**	0.3 (0.001)	**0.18 (0.002)**
	D1D2D3	**0.35 (0.001)**	0.14 (0.001)	0.34 (0.002)	0.27 (0.004)	0.33 (0.001)	0.17 (0.002)

Table S17. Weighted ROC-AUC for prediction of diagnosis at discharge (DD) in the *training and validation* dataset and in the *hold-out test* dataset. Related to Table 2

Training and validation			
INPUT VARIABLES		RF	
		MVTS	STATES
NO PDA	D1	0.63 (0)	0.58 (0)
	D2	0.63 (0)	0.59 (0)
	D3	0.62 (0)	0.59 (0.001)
	D1D2	0.64 (0)	0.59 (0)
	D2D3	0.64 (0)	**0.6 (0)**
	D1D2D3	**0.65 (0)**	**0.6 (0)**
Hold-out test			
INPUT VARIABLES		RF	
		MVTS	STATES
NO PDA	D1	0.732 (0)	0.54 (0.001)
	D2	0.696 (0)	0.532 (0.001)
	D3	0.686 (0)	0.537 (0.001)
	D1D2	0.738 (0)	0.537 (0.001)
	D2D3	0.715 (0)	0.539 (0.001)
	D1D2D3	**0.748 (0)**	**0.541 (0.001)**

Table S18. Weighted precision, recall and F1-score for prediction of diagnosis at discharge (DD) in the *training and validation* dataset and in the *hold-out test* dataset. Related to Table 2

Training and validation							
INPUT VARIABLES		RF		RF		RF	
		Precision		Recall		F1-score	
		MVTS	MV TS	MVTS	STATES	MVTS	STATES
NO PDA	D1	0.4 (0.001)	0.32 (0.001)	**0.38 (0.001)**	0.47 (0.003)	0.38 (0.001)	0.36 (0.001)
	D2	0.38 (0.001)	0.33 (0.001)	**0.38 (0.001)**	**0.48 (0.002)**	0.38 (0.001)	0.37 (0.001)
	D3	0.38 (0.001)	0.33 (0.001)	0.37 (0.001)	0.47 (0.003)	0.37 (0.001)	0.37 (0.001)
	D1D2	**0.42 (0.001)**	**0.34 (0.001)**	0.37 (0.001)	0.47 (0.002)	**0.39 (0.001)**	0.37 (0.001)
	D2D3	0.4 (0.001)	**0.34 (0)**	0.37 (0.001)	0.47 (0.002)	0.38 (0.001)	0.37 (0.001)
	D1D2D3	**0.42 (0.001)**	**0.34 (0.001)**	0.36 (0.001)	0.47 (0.002)	0.38 (0.001)	**0.38 (0.001)**
Hold-out test							
INPUT VARIABLES		RF		RF		RF	
		Precision		Recall		F1-score	
		MVTS	MV TS	MVTS	STATES	MVTS	STATES
NO PDA	D1	0.291 (0.001)	0.151 (0.003)	**0.386 (0.001)**	0.271 (0.007)	0.323 (0.001)	0.165 (0.004)
	D2	0.265 (0.002)	0.115 (0.002)	0.338 (0.002)	0.298 (0.005)	0.29 (0.002)	0.159 (0.003)
	D3	0.274 (0.003)	0.125 (0.002)	0.281 (0.003)	**0.315 (0.007)**	0.271 (0.002)	0.175 (0.003)
	D1D2	0.324 (0.001)	0.141 (0.002)	0.369 (0.001)	0.26 (0.005)	**0.336 (0.001)**	0.161 (0.002)
	D2D3	0.319 (0.002)	0.127 (0.002)	0.299 (0.001)	**0.317 (0.004)**	0.298 (0.001)	**0.176 (0.002)**
	D1D2D3	**0.346 (0.001)**	**0.139 (0.001)**	0.335 (0.002)	0.273 (0.004)	0.332 (0.001)	0.17 (0.002)
